# *DPC29* promotes post-initiation mitochondrial translation in *Saccharomyces cerevisiae*

**DOI:** 10.1093/nar/gkac1229

**Published:** 2023-01-09

**Authors:** Kyle A Hubble, Michael F Henry

**Affiliations:** Department of Molecular Biology, Rowan University School of Osteopathic Medicine, Stratford, NJ 08084, USA; Graduate School of Biomedical Sciences, Rowan University, Stratford, NJ 08084, USA; Department of Molecular Biology, Rowan University School of Osteopathic Medicine, Stratford, NJ 08084, USA; Graduate School of Biomedical Sciences, Rowan University, Stratford, NJ 08084, USA

## Abstract

Mitochondrial ribosomes synthesize essential components of the oxidative phosphorylation (OXPHOS) system in a tightly regulated process. In the yeast *Saccharomyces cerevisiae*, mitochondrial mRNAs require specific translational activators, which orchestrate protein synthesis by recognition of their target gene's 5'-untranslated region (UTR). Most of these yeast genes lack orthologues in mammals, and only one such gene-specific translational activator has been proposed in humans—TACO1. The mechanism by which TACO1 acts is unclear because mammalian mitochondrial mRNAs do not have significant 5'-UTRs, and therefore must promote translation by alternative mechanisms. In this study, we examined the role of the TACO1 orthologue in yeast. We found this 29 kDa protein to be a general mitochondrial translation factor, Dpc29, rather than a *COX1*-specific translational activator. Its activity was necessary for the optimal expression of OXPHOS mtDNA reporters, and mutations within the mitoribosomal large subunit protein gene *MRP7* produced a global reduction of mitochondrial translation in *dpc29*Δ cells, indicative of a general mitochondrial translation factor. Northern-based mitoribosome profiling of *dpc29*Δ cells showed higher footprint frequencies at the 3' ends of mRNAs, suggesting a role in translation post-initiation. Additionally, human TACO1 expressed at native levels rescued defects in *dpc29*Δ yeast strains, suggesting that the two proteins perform highly conserved functions.

## INTRODUCTION

OXPHOS (oxidative phosphorylation) activity is central to cellular metabolism and mitochondrial function, therefore the generation of these biological complexes is tightly regulated. The electron transport chain consists of protein complexes whose subunits are of dual origin (1). The nuclear genome-encoded components are ultimately transported across the mitochondrial double membrane, while mitochondrial DNA (mtDNA)-encoded genes are expressed within the organelle. These processes are carefully balanced, as mismanagement or overproduction of these catalytic core enzymes results in oxidative stress, DNA damage and apoptosis (2,3).

Mitochondria possess their own transcriptional and translational machinery to express the mtDNA-encoded proteins (4). Like their ancient prokaryote ancestors, mitochondrial gene expression is thought to be a membrane-associated, coupled process (5). While much of gene expression within mitochondria resembles that of their bacterial ancestors, its divergent evolution from prokaryotes has allowed for many novel features. For instance, mitochondrial transcription is performed by a single subunit bacteriophage-type RNA polymerase along with one general transcription factor (6,7). Additionally, the mitoribosome is highly specialized for the synthesis of hydrophobic membrane proteins of the electron transport chain, which are co-translationally inserted into the mitochondrial inner membrane (8).

Whereas nuclear genes are predominantly transcriptionally controlled, mitochondrial gene expression is regulated at the translational level. In baker's yeast, factors called ‘translational activators’ are rate limiting and gene specific (9). These activators have been defined as membrane-tethered factors that promote translation initiation by binding either the elongated 5'-untranslated regions (UTRs) of their target mRNAs or components of the mitoribosome. Although their molecular mechanisms are not well understood, *in vitro* RNA binding and mitoribosome co-purification experiments suggest that they recognize 5'-UTR secondary structure and recruit mRNAs to the translation apparatus (10,11). In addition, some genes possess multiple translational activators, which can cooperate in feedback loops to control translational flux (12–14).

Historically, *Saccharomyces cerevisiae* has served as the primary model organism for studying mitochondrial gene expression due to its inherent advantages. These include the ability (i) to maintain cells on fermentative media in the absence of a functioning respiratory chain, (ii) to genetically modify the mitochondrial genome and (iii) to perform relatively fast and inexpensive genetic screens. Although mitochondrial gene expression in baker's yeast and mammals have differences, they share many common features.

Since mammalian mitochondrial mRNAs generally lack 5'-UTRs, they presumably activate translation by alternative mechanisms. While mammalian mitoribosomes preferentially select the start codon of leaderless mRNAs (15), the question remains of whether gene-specific translational activators are required for fidelity of translation initiation. As expected, most of the characterized yeast translational activators lack mammalian orthologues. However, one gene-specific ‘translational activator’ for the mammalian cytochrome *c* oxidase subunit I (*COX1*) mRNA has been reported—TACO1 (16,17). Point mutations in this gene cause mitochondrial dysfunction and late-onset Leigh syndrome in humans and mice (16–18). Although previous *in vitro* experiments found that the mouse TACO1 protein binds *COX1* mRNA, the cellular consequences of disrupting this interaction were not established (17). Interestingly, *COX1* mRNA–mitoribosome binding in mice was maintained following the loss of TACO1, leading to the proposal that the TACO1 protein acts at a translational stage after mRNA–mitoribosome binding (17). Interestingly, yeast cells express a *TACO1* orthologue, termed *DPC29* (*YGR021W*) (19), which shares 29% identity and 43% similarity with the human protein.

In this study, we further define the role of TACO1 in mitochondrial translation by investigating its yeast orthologue. We report that Dpc29 (Delta-Psi dependent mitochondrial import and Cleavage protein of 29 kDa) plays a general role in post-initiation mitochondrial translation. Both mammalian and yeast cells lacking TACO1/Dpc29 produce less Cox1 and have reduced cytochrome *c* activity, but still respire. However, Dpc29 activity is not gene specific as its loss affects the expression of all mitochondrial-encoded OXPHOS reporter genes. Furthermore, *dpc29*Δ strains harbouring mutations within the Mrp7 mitoribosomal large subunit protein, which are respiratory synthetic lethal, show a global reduction of mitochondrial translation. Consistent with this role, Dpc29 directly binds specific mitoribosomal proteins during active translation in cells. Mitoribosome profiling in *dpc29*Δ cells revealed that footprint distribution of the mitochondrial-encoded transcripts was unchanged at the 5' end, indicating that translation initiation was unaffected. Finally, we show that human TACO1 expression in yeast rescues *dpc29*Δ *mrp7* respiratory synthetic lethality and mtDNA reporter expression in *dpc29*Δ cells. Thus, further studies of *DPC29* in yeast should be generally applicable to human *TACO1*.

## MATERIALS AND METHODS

### Strains, media and genetic methods

Yeast strains used in this study are listed in Supplementary Table S1. All strains are derived from either NB40-36a or BMA64-1A. Cells were grown at 30°C in rich medium (YP) containing 1% yeast extract and 2% peptone or minimal medium containing 0.17% yeast nitrogen base, 0.5% ammonium sulphate and the appropriate amino acids, unless otherwise indicated. Media contained 2% glucose, galactose, lactate, ethanol or 3% glycerol, as indicated. The rich or minimal ‘D’ medium used for the respiratory synthetic lethal screen contained 3% glycerol, 2% ethanol and 0.2% glucose as carbon sources. The *mrp7-1* (g77a), *mrp7-2* (g130a), *mrp7-3* (c125t) and *mrp7-4* (c125a) synthetic respiratory lethal mutations were re-engineered by site-directed mutagenesis and integrated into the yeast chromosome using the yeast integrative vector Yip5 (20–22). Dpc29 was tagged with TWINSTREP at the C-terminus (23). The sequence of this fusion protein is provided in Supplementary Table S3.

### Submitochondrial localization

Purification of yeast mitochondria was performed as previously described (24), and 200 μg aliquots were converted to mitoplasts by incubation in ice-cold hypotonic buffer: 20 mM HEPES-KOH pH 7.4. For detection of peripheral inner membrane interactions, mitoplasts were incubated with increasing salt conditions, i.e. 100, 250 and 500 mM KCl, and sonicated 3× 30 s at a 40% duty cycle (Branson 450). For detection of integral membrane proteins, mitoplasts were resuspended in 100 mM Na_2_CO_3_ and placed on ice for 30 min. For all conditions tested, membranes were pelleted at 100 000 *g* at 4°C for 30 min using a TLA55 rotor. Both pellet and trichloroacetic acid (TCA)-precipitated supernatant proteins were resuspended in equal volumes of Laemmli buffer prior to gel loading (25). Western blot analysis was performed as described previously (26). Percent Dpc29 per fraction was calculated as a percentage of the sum of pellet (P) plus supernatant (S). To distinguish mitochondrial inner membrane space from matrix proteins, mitoplasts were incubated with 50 μg/ml proteinase K for 25 min on ice. Anti-Dpc29 polyclonal antibody was obtained from Covance Inc. (Denver, PA, USA) by immunizing rabbits with recombinant full-length glutathione *S*-transferase (GST)–Dpc29 purified from *Escherichia coli*. Anti-Cox2 mouse monoclonal antibody (mAb; 4B12A5) was obtained from Abcam. Anti-Arg8 rabbit polyclonal antibody was a gift from Tom Fox at Cornell University (27). Anti-Cyc1 rabbit polyclonal antibody was a gift from Antoni Barrientos at University of Miami (28).

### Labelling of mitochondrial translation products *in vivo* and *in organello*

*In vivo* radiolabelling of mitochondrial translation products was performed as previously described (24). To test inhibition of mitochondrial translation, tigecycline was added at 4 mg/ml alongside cycloheximide for 5 min prior to the addition of [^35^S]cysteine and [^35^S]methionine (29).

*In organello* radiolabelling of isolated mitochondria was performed as previously described (30).

### Quantitative analysis of mitochondrial protein levels

To quantify respiratory chain subunit proteins, western blot analysis was performed in biological triplicate with mitochondrial protein extracts using IRDye 800CW goat anti-mouse and anti-rabbit secondary antibodies (LI-COR) imaged on a Typhoon scanner. Revert™ total protein stain (LI-COR) was used for western blot normalization. Respiratory chain complexes from 10 μg of *n*-dodecyl-β-d-maltoside (DDM)-solubilized yeast mitochondria were resolved using blue native polyacrylamide gel electrophoresis (BN-PAGE; Invitrogen BN1001) according to the manufacturer's protocols. Proteins were transferred to a polyvinyldifluoridene (PVDF) membrane (Millipore Immobilon-FL), then western blot analysis was performed and the proteins were quantified by the same method as for the subunit proteins. Primary antibodies used were anti-Cox1 mouse monoclonal antibody (mAb; 1D6E1A8), anti-Cox2 mouse mAb (4B12A5), anti-Cox3 mouse mAb (DA5BC4) obtained from Abcam, anti-Cob rabbit polyclonal (97505)—a gift from Brian Robinson (31), anti-Atp2 rabbit polyclonal—a gift from Alexander Tzagoloff (32) and anti-Atp6 rabbit polyclonal—a gift from Jean Velours (33).

### RNA isolation and qRT-PCR

For RNA extraction, cells were grown to exponential phase in rich galactose medium and RNA was isolated using TRIzol RNA extraction reagent (Ambion) according to the manufacturer's instructions. RNA from the mitoribosome purification was isolated using the Direct-zol RNA MiniPrep kit (Zymo Research). cDNA synthesis and quantitative real-time polymerase chain reaction (qRT-PCR) was performed in triplicate using the Luna qRT-PCR kit (New England Biolabs) and the Q–qPCR instrument and software (QuantaBio) according to the manufacturer's recommendations. Primer sequences are listed in Supplementary Table S2. *ACT1* was used to normalize mRNA analysis for whole cells, while *21S* rRNA was used for mitoribosome occupancy analysis.

### Site-specific photocross-linking

Dpc29 protein interactions were determined by *in vivo* photocross-linking as previously reported (34) with several modifications. Dpc29 was C-terminally tagged with TWINSTREP, affinity-purified using MagStrep ‘type3’ XT beads and eluted using Streptactin buffer E (Iba-lifesciences). To screen potential cross-linking interactants, western blot analysis was performed using anti-Dpc29 rabbit polyclonal antibody (this study). For liquid chromatography/tandem mass spectrometry (LC-MS/MS) identification of cross-linked partners, the purification was scaled up 6-fold, using 300 ml of exponential phase YP-Gal yeast culture. Eluates were resolved by sodium dodecylsulphate (SDS)–PAGE and Coomassie stained. Bands of interest were excised, and proteins identified by LC-MS/MS (Wistar Institute, Philadelphia, PA, USA). The raw LC-MS/MS data are provided in Supplementary Table S4. When inhibiting mitochondrial translation, tigecycline was added to cultures at 4 mg/ml at 1 h prior to cross-linking.

Since site-directed photocross-linking limits cross-links to a single Dpc29 residue, the chance of identifying false peptides formed from the cross-link junction is low. To further ensure proper protein identification, we only examined proteins in a gel slice corresponding to the molecular weight of the cross-linking complex and limited our candidate proteins to those with molecular weights equal to the difference between the complex and Dpc29–TWINSTREP bait. Furthermore, proteins with a single unique peptide identified by MS were designated as low confidence and not considered.

### *DPC29* respiratory synthetic lethal screen

This genetic screen was performed as previously reported with several modifications (24). The parental strain is deleted for *DPC29* but harbours the same *URA3 ADE3* plasmid containing *DPC29*. Ethyl methanesulphonate (EMS) was used to generate *mrp7-1* and *mrp7-2* synthetic respiratory lethal mutants while UV-induced mutagenesis produced the *mrp7-3* and *mrp7-4* alleles (Stratalinker 1800, Stratagene). The exposure required for UV-induced mutagenesis was determined empirically to achieve 50% killing.

### Sucrose gradient sedimentation of mitoribosomes

Mitoribosomes from mitochondrial extracts were sedimented and analysed as previously described (24).

### Cytochrome *c* oxidase activity assay

Cytochrome *c* oxidase activity of purified mitochondria was determined using the Cytochrome *c* Oxidase Assay (ScienCell) according to the manufacturer's protocols. Activity was normalized to total mitochondrial protein added per reaction.

### Mitoribosome occupancy and profiling

To determine overall mRNA occupancy on mitoribosomes, a previously reported protocol for mitoribosome profiling was followed with several modifications (35). To preserve full-length mRNAs, the use of RNase was omitted prior to purification of mitoribosomes tagged with MrpS17-3×FLAG. Following cryogenic lysis of actively growing yeast cells, anti-FLAG purification of mitoribosomes was performed (MonoRab™ Anti-DYKDDDDK Magnetic Beads, GenScript), mRNA was purified and qRT-PCR analysis was performed. For mitoribosome profiling, footprints were produced with RNase If (NEB) prior to mitoribosome anti-FLAG immunoprecipitation. RNA isolation and northern analysis were performed as previously reported (35).

### Statistical analysis

All quantitative experiments were performed in triplicate. Error bars represent the mean ± standard error (SE). Statistical tests were determined using an unpaired two-tailed Student's *t*-test utilizing GraphPad Prism 9. Significant difference in means is indicated as: *P <0.05, **P <0.01, ***P <0.001 and ****P <0.0001.

### Software

Protein 3D models were generated using PyMOL. Bar graphs were generated using GraphPad Prism 9. Predictive models of TACO1 orthologues were generated using Phyre2 software. Image Studio Lite was used for quantification of Typhoon scans of western and radiolabelled experiments.

## RESULTS

### Deletion of the yeast *TACO1* orthologue, *DPC29*, reduces Cox1 protein levels

**Figure 1.**
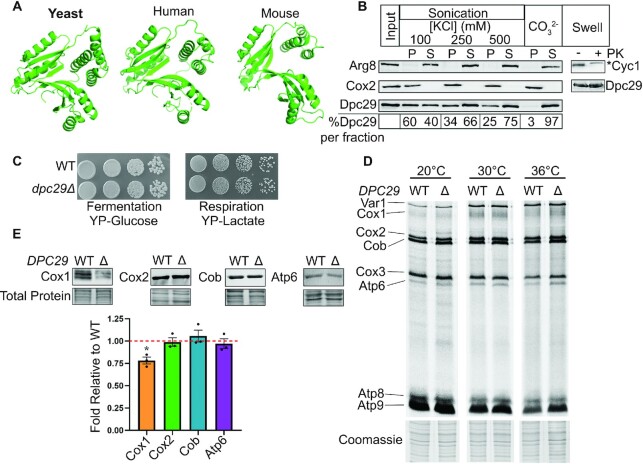
Loss of the yeast *TACO1* orthologue *DPC29* reduces Cox1 protein levels. (**A**) Predictive protein models of yeast and human TACO1 produced using the PHYRE2 (33) protein fold recognition server with the mouse TACO1 crystal structure (PDB 5EKZ) as a reference. Images generated with PyMol. (**B**) Western analysis of Dpc29 submitochondrial localization and inner membrane association. Isolated mitochondria were either untreated (left-most lane), sonicated with KCl (middle left lanes), treated with carbonate (middle right lanes) or resuspended in hypotonic buffer (right-most two lanes) with (+) or without (–) proteinase K treatment (PK). Pellet (P), supernatant (S) or whole mitochondrial lysate fractions were resolved by 10% SDS–PAGE and probed with antisera (indicated on the right or left of each panel). The asterisk denotes an anti-Cyc1 antibody-cross-reactive band. Dpc29 levels were measured using Image Studio, and the percentage of Dpc29 per fraction was calculated as a fraction of the sum of P + S signal averaged over three biological replicate experiments. (**C**) Growth of wild-type (WT) and *dpc29*Δ cells. Mid-log cells were spotted as 8-fold serial dilutions on fermentative (YP-Glucose) or respiratory (YP-Lactate) media and incubated at 30°C for 3 days. (**D**) Mitochondrial translation profiles. WT and *dpc29Δ* mid-log cells grown in galactose media were pulse-labelled with [^35^S]methionine and [^35^S]cysteine for 10 min in the presence of cycloheximide at 20, 30 and 36°C. Proteins were resolved by SDS–PAGE, stained and visualized by autoradiography. A segment of corresponding Coomassie stain is shown below each lane. (**E**) Quantification of representative mitochondrial-encoded OXPHOS proteins. Isolated mitochondria from mid-log cells grown in galactose were solubilized, and select OXPHOS complex subunits were examined by western analysis (upper panels) and normalized to total protein loaded (bottom panels). Bar chart showing normalized levels of Cox1, Cox2, Cob and Atp6. Error bars represent the SE from three biological repeats, and the asterisk indicates a *P*-value <0.05 (see the Materials and Methods for more information on statistical analysis).

TACO1 is reported to act as a cytochrome *c* oxidase subunit 1 (Cox1)-specific translational activator whose loss results in decreased Cox1 protein levels in cells and late-onset Leigh syndrome in humans and mice (16–18). An *S. cerevisiae* TACO1 orthologue, Dpc29, shares 29% identity and 43% similarity with human TACO1 (Supplementary Figure S1A). Predictive structural models of the yeast and human proteins show striking structural similarity despite differences in amino acid identity (Figure 1A) (17,36). Furthermore, like mammalian TACO1, its yeast orthologue localizes to the mitochondria and peripherally associates with the matrix side of the mitochondrial inner membrane, where the translational apparatus localizes (Figure 1B). Given this conservation, we anticipated that the human and yeast orthologues would have the same mitochondrial role and thus employed the genetically amenable yeast model to define its mechanism of action.

Studies of yeast *DPC29* have been challenging because deletion mutants lack a discernible growth phenotype. Yeast Cox1 protein steady-state levels were modestly reduced (Figure 1E) but did not impair respiratory growth (Figure 1C). In the human and mouse models, loss of TACO1 lowered Cox1 levels more severely in some tissues; however, overall respiratory capacity was sufficient for embryogenesis and survival to adulthood (16,17). Reportedly, this was due to a Cox1-specific translational defect. However, this defect was absent in *dpc29Δ* yeast cells as determined by *in vivo* and *in organello* mitochondrial translation assays at all pulse durations and temperatures tested (Figure 1D; Supplementary Figure S1C). Thus, Cox1 translation appears unchanged but mitochondrial steady-state levels are reduced. This result is not due to Cox1 instability as shown by pulse–chase analysis (Supplementary Figure S1B). One interpretation of these results is that modest translational defects could be masked due to the cycloheximide treatment that affects cellular protein homeostasis during the *in vivo* labelling assay (37). While *in organello* translation assays are performed without cycloheximide treatment, this approach uses mitochondria removed from the cellular environment which may also limit translational rates. We propose that *dpc29*Δ mitochondrial translation defects are only observed under physiological conditions.

### Membrane-directed translation of soluble reporter proteins is diminished in *dpc29*Δ cells

**Figure 2.**
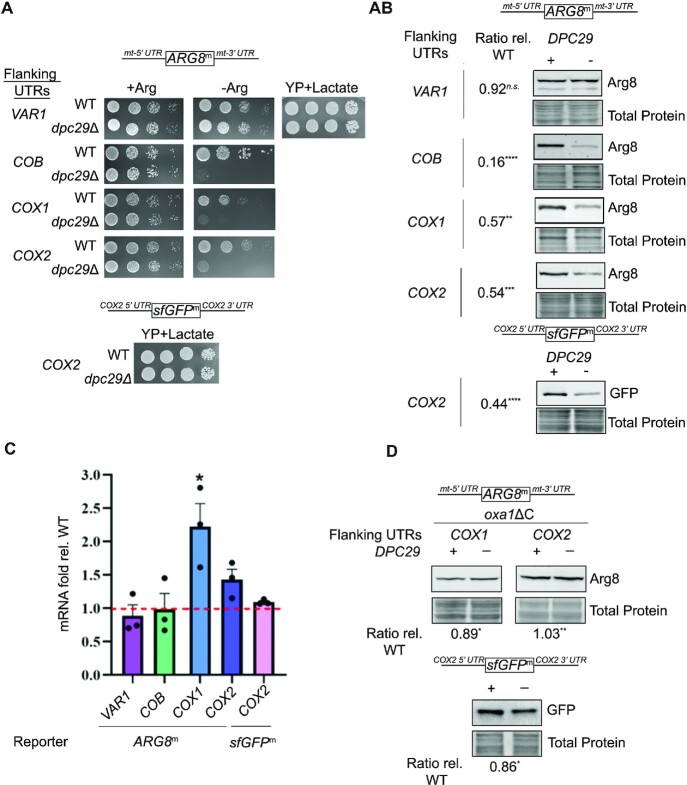
Membrane-directed translation of soluble reporter proteins is diminished in *dpc29Δ* cells. (**A**) Growth analysis of mitochondrial translation reporter strains. Mid-log cells were spotted as 8-fold serial dilutions on synthetic media containing (+Arg) or lacking (–Arg) arginine and incubated at 30°C for 3 days. The two respiratory competent strains were also spotted on rich media containing lactate (YP + Lactate). (**B**) Steady-state levels of mitochondrial reporter proteins during growth on galactose. Mitochondrial-recoded Arg8 (upper blots) or sfGFP (lower blot) proteins from whole-cell extracts were separated by 10% SDS–PAGE, stained and analysed by western blotting (upper panels) with either Arg8 or GFP antisera (indicated on the right), and normalized to total protein loaded (below each blot). Each blot is representative of three biological replicates. Ratios of Arg8^m^ relative to the WT were calculated and are shown directly to the left of each blot. (**C**) Steady-state reporter mRNA levels relative to the WT from (B) were measured by qRT-PCR using primers within either *ARG8*^m^ or *sfGFP*^m^ coding sequences. Individual experimental values are indicated with filled circles, and error bars represent the SE. (**D**) The effect of *oxa1*ΔC on reporter expression in *dpc29*Δ cells. Arg8^m^ and sfGFP^m^ levels in *oxa1*ΔC and *oxa1*ΔC *dpc29*Δ cells were measured by western analysis as described in (B). One, two, three and four asterisks indicate *P*-values <0.05, 0.01, 0.001 and 0.0001, respectively. For (A), (B) and (D), graphical representations of the *ARG8*^m^ and *sfGFP*^m^ reporter mRNAs with flanking mitochondrial 5′- and 3′-UTRs are shown above their respective blot or spotting images.

Our use of translational reporter genes as an alternative approach to examine protein expression in *dpc29Δ* cells provided the first evidence that *DPC29* plays a general role in mitochondrial translation and alleviates the translational stress of expressing these soluble proteins at membrane-directed mitoribosomes. When the nuclear-encoded *ARG8* gene is recoded to the mitochondrial genetic code (*ARG8*^m^) (12,27,38) and used to replace mitochondrial encoded open reading frames (ORFs), its expression becomes independent of the post-translational stability of the replaced gene. Additionally, *in vivo* mitochondrial translation can be assayed without inhibiting cytosolic translation with cycloheximide. When the ORFs of representative mitochondrial protein-coding genes (*COB*, *COX1*, *COX2* and *VAR1*) were replaced with the *ARG8^m^* sequence, expression of the resulting reporter mRNAs was unaffected in *dpc29Δ* cells (Figure 2C). However, the loss of Dpc29 strongly decreased cell growth on media lacking arginine (Figure 2A) and reduced *ARG8*^m^ reporter protein steady-state levels for all the mitochondrial-encoded genes assayed except *VAR1* (Figure 2B). The reduced Arg8^m^ levels are not due to protein stability because its translation was also reduced in a radiolabelling pulse (Figure 8D, right panel lanes 1 and 2; Supplementary Figure S3C, lanes 1 and 2). These results contrasted with our previous experiments where *dpc29Δ* cells showed normal *in vivo* mitochondrial translation and a modest decrease in Cox1 steady-state protein levels (Figure 1D, E). One explanation for this discrepancy is that the OXPHOS 5′ and 3′ sequences (*COB*, *COX1* and *COX2*) target the *ARG8*^m^ mRNA to locations where hydrophobic respiratory chain proteins that normally emerge from mitoribosomes are directed toward the mitochondrial inner membrane to facilitate complex assembly. We propose that synthesis of the soluble mitochondrial matrix protein Arg8 toward the inner membrane at these locations may result in steric hindrance, and Dpc29 becomes necessary for optimal translation during these experimental conditions. Consistent with this idea, Var1 reporter expression is Dpc29 independent because Var1-expressing mitoribosomes are thought to translate at distinct mitoribosome assembly sites that direct their products to the matrix (39). This occurs because the *VAR1* product, like Arg8, is a soluble matrix protein.

To show that the OXPHOS reporter translation defects observed were not *ARG8*^m^ specific, a mitochondrial-recoded superfolder green fluorescent protein (*sfGFP*^m^) reporter flanked by *COX2* 5′- and 3′-UTRs was also assayed (37). Consistent with our previous results, this reporter exhibited *DPC29-*dependent expression (Figure 2B). Given these results, we predicted that relaxing the membrane–mitoribosome interaction at OXPHOS assembly regions would resolve the steric hindrance encountered by soluble reporter proteins translated at these sites and reduce *DPC29* dependency. To test this hypothesis, we deleted the C-terminus of Oxa1, one of three mitoribosome membrane-anchoring sites (8,40–43). In *oxa1ΔC* cells, OXPHOS translation products are directed to the matrix rather than the mitochondrial inner membrane (40). When the *oxa1ΔC* mutation was introduced into *dpc29Δ* cells expressing either *cox1*Δ::*ARG8*^m^, *cox2*Δ::*ARG8*^m^ or *cox2*Δ::*sfGFP*^m^, reporter levels were restored (Figure 2D).

In summary, these results indicate that the function of *DPC29* in mitochondrial translation is not gene specific. In addition, the translation of soluble proteins towards the hydrophobic mitochondrial inner membrane generates translational stress requiring *DPC29* function for optimal expression. While we suggest that the stress is due to steric hindrance, an alternative explanation is that these reporter sequences contain translational pause sites whose effects are amplified for mitoribosomes facing the membrane, which are regulated by different translation factors.

### Dpc29 binding to mitoribosomal proteins requires actively translating cells

**Figure 3.**
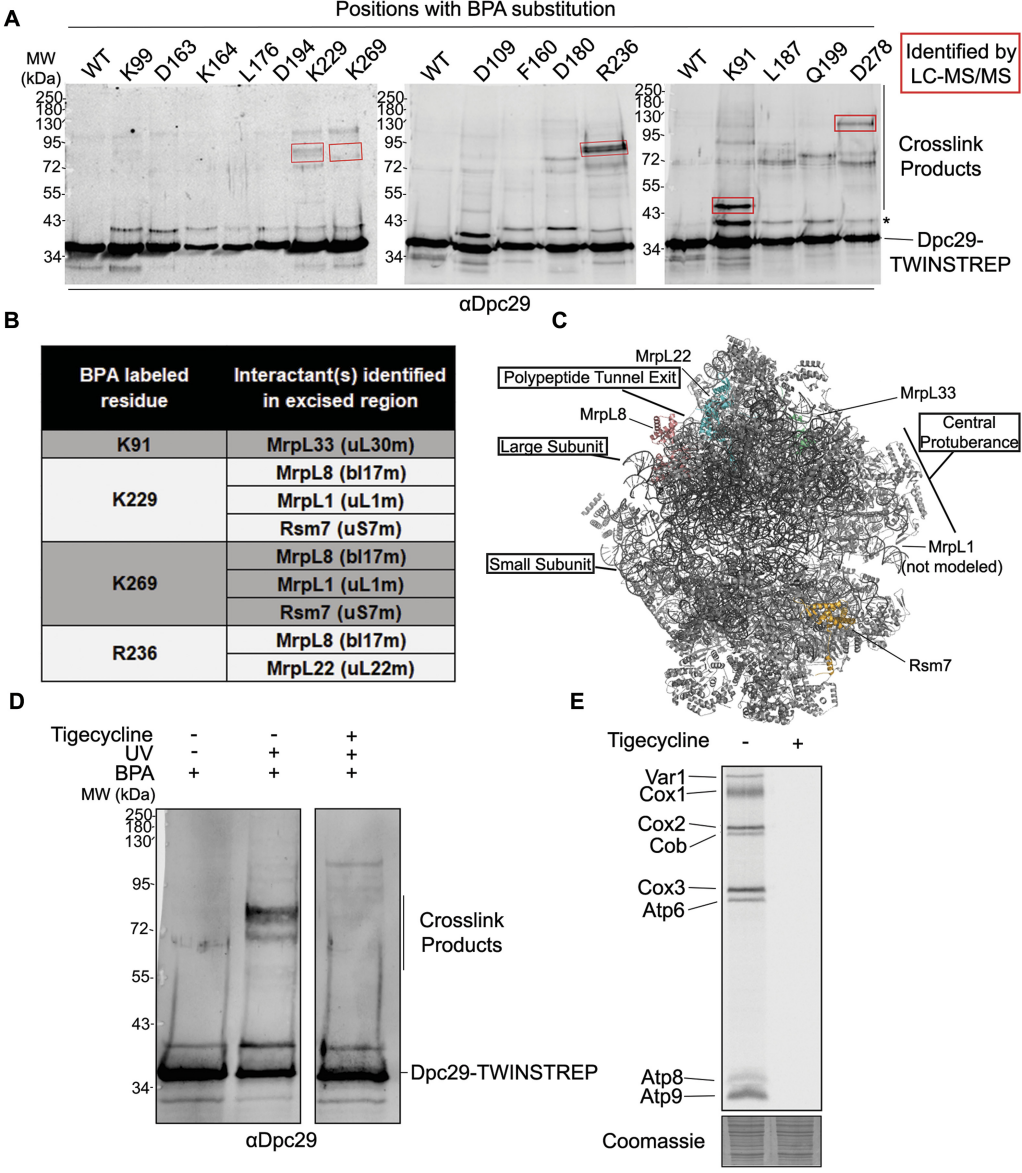
Dpc29–mitoribosome binding requires active translation. (**A**) Western analysis of Dpc29–TWINSTREP BPA-cross-link complexes affinity-purified from cells post-UV treatment. Cells containing plasmids expressing Dpc29–TWINSTREP with BPA substituted at the amino acid positions indicated above the blot were grown in galactose media. Molecular weights are indicated in kDa on the left. Red boxes show regions excised from large-scale purification repeats and subjected to LC-MS/MS identification. The asterisk denotes the probably uncross-linked Dpc29–TWINSTREP that has retained its mitochondrial targeting sequnce (MTS). (**B**) Dpc29 direct interactants identified by LC-MS/MS. Non-mitochondrial or mature proteins with predicted molecular weights outside the range for possible interactants were excluded from the list as contaminants. This data represent one single LC-MS/MS analysis per BPA mutant. (**C**) Yeast mitoribosome image (PDB 5MRC) with Dpc29 direct interactants coloured. Although MrpL1 was not modelled in this structure, it is known to be a component of the large subunit central protuberance. (**D**) K229–BPA cross-linking in the absence of active mitochondrial translation. The Dpc29–TWINSTREP K229–BPA mutant cells were treated with 4 mg/ml tigecycline for 1 h, UV cross-linked, and affinity-purified Dpc29–TWINSTREP K229–BPA complexes were subjected to western analysis. Images were captured from the same blot with middle lanes excluded. (**E**) Mitochondrial translation profiles of cells grown to mid-log in galactose and pulse-labelled with [^35^S]methionine and [^35^S]cysteine in the presence of cycloheximide with and without tigecycline. Proteins were resolved by SDS–PAGE, stained, and visualized by autoradiography. A segment of corresponding Coomassie stain is shown below each lane.

If Dpc29 acts during mitochondrial translation, we reasoned that it might physically interact with components of the mitochondrial protein synthesis machinery. However, co-immunoprecipitation experiments using either whole-cell or mitochondrial lysates did not detect Dpc29 interactants (our unpublished results). Since mitoribosome translation is disrupted in lysates, we employed site-directed photocross-linking (44) to capture transient interactions with actively translating mitochondria in live cells (34). A cross-linking approach was also required to capture mouse TACO1 interactors (17). Dpc29 was C-terminally tagged with TWINSTREP (Dpc29–TWINSTREP) and found to functionally complement untagged Dpc29 (Supplementary Figure S3A). Dpc29–TWINSTREP variants were created by changing select codons to an amber stop codon. These variants were then introduced into *dpc29Δ* yeast cells containing the aminoacyl-synthetase/tRNA pair to permit amber codon suppression via incorporation of the phenylalanine analogue 4-benzoylphenyl-pentanoic acid (BPA) at these sites. Supplementing the growth medium with pBpa enabled incorporation of this amino acid at the desired position and the formation of zero-length specific cross-linked products upon UV irradiation (Figure 3A). Fifteen predicted Dpc29 surface residues were chosen (Figure 3A), exponential phase cells were irradiated, cross-link products were purified under denaturing conditions via the C-terminal TWINSTREP tag on Dpc29 and proteins were resolved by SDS–PAGE. Western blotting against Dpc29 showed multiple BPA-specific cross-link products of different sizes (Figure 3A). Concerned with detection, we limited our analysis to positions K91^pBpa^, K229^pBpa^, R236^pBpa^, K269^pBpa^ and D278^pBpa^, which showed the strongest unique cross-link bands. The cross-linking experiment was scaled-up for these five BPA mutants and the corresponding gel regions excised for LC-MS/MS analysis.

Dpc29 directly cross-linked to five mitoribosomal subunit proteins. When mapped to the surface of the mitoribosome model, four are components of the large subunit while one is found in the small subunit. Two (MrpL22 and MrpL8) of the large subunit proteins are found at the polypeptide tunnel exit and face the mitochondrial inner membrane, while the other two (MrpL1 and MrpL33) face the matrix (Figure 3B, C). Rsm7 is a mitoribosome small subunit protein (Figure 3B, C). Several BPA-labelled residues within Dpc29 cross-link with multiple mitoribosomal proteins, suggesting that Dpc29 interacts with different regions of the mitoribosome (Figure 3B, C). These results are consistent with the recent BioID study where Dpc29 fused to a biotin ligase (BirA*) biotinylated mitoribosomal subunit proteins, indicating their proximity (45). However, these two approaches are not expected to identify the same proteins within the mitoribosome as the labelling radius for BioID is 100 Å (approximately the diameter of the mitoribosome large subunit) (8), while the site-directed photocross-linking employed in this study is zero-length.

To confirm that Dpc29–mitoribosome interactions were translation dependent, the cross-linking experiment was repeated with the addition of the mitoribosome-specific translational inhibitor tigecycline. This potent antibiotic rapidly inhibits mitochondrial translation in actively growing yeast cells (29). K229^pBpa^ cross-linking products were absent following tigecycline treatment that fully inhibited mitochondrial translation (Figure 3D, E). This result demonstrates that Dpc29 binding to mitoribosomes requires actively translating cells.

### Mitochondrial translation is severely reduced in *dpc29*Δ cells containing N-terminal point mutations in the mitoribosomal LSU protein Mrp7 (bL27)

In mouse and human cells, the loss of *TACO1* causes severe defects in Cox1 protein levels, resulting in late-onset Leigh syndrome (16,17). Although yeast cells lacking the *TACO1* orthologue *DPC29* have reduced Cox1 protein, these cells still respire normally. Therefore, we reasoned that *DPC29* function could be redundant, or its loss could be compensated for, masking a more severe phenotype. To investigate this, we utilized the phenomenon of respiratory synthetic lethality (24). This approach assumes that if another gene compensates for *DPC29* activity, then *dpc29*Δ cells bearing a mutation in the product will fail to respire. Characterization of these respiratory lethal mutants could provide insight regarding *DPC29* function.

**Figure 4.**
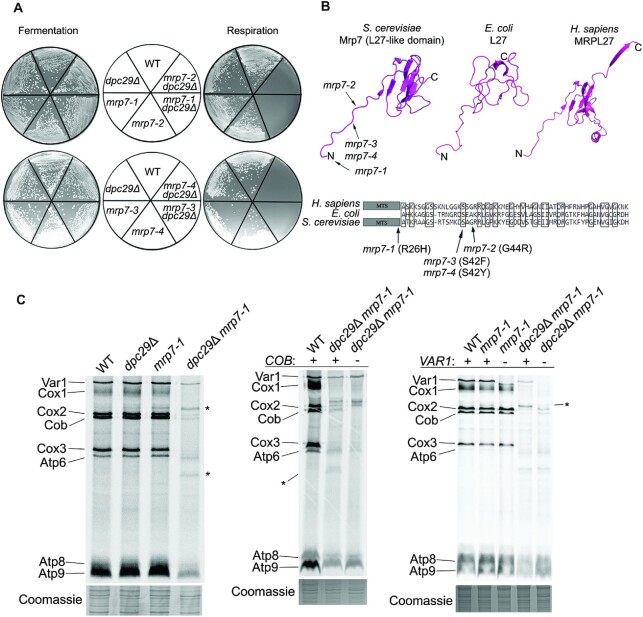
N-terminal point mutations in *MRP7* are respiratory synthetic lethal with a *DPC29* deletion. (**A**) Fermentative and respiratory growth of respiratory synthetic mutants. Mutants were streaked to either YP + glucose (fermentative) or YP + lactate (respiratory) media as indicated by the centre plates and incubated for 3 days at 30°C. (**B**) Comparison of *E. coli* L27 and *H. sapiens* MRPL27 with the *S. cerevisiae* L27-like domain with respiratory synthetic lethal mutations indicated. Yeast Mrp7 residues 28–108 (PDB 5MRC), *E. coli* L27 (PBD 4YBB) and *H. sapiens* MRPL27 (PDB 3J9M) were generated from PyMol. The *mrp7-1* mutation (R26H) is at the –1 position of the mature protein sequence. Clustal Omega alignments (EMBL-EBL) of the three proteins were produced and the N-terminal regions are shown below. (**C**) Mitochondrial translation profiles of respiratory synthetic lethal mutants. Cells were grown to mid-log phase in galactose and pulse-labelled with [^35^S]methionine and cysteine in the presence of cycloheximide. Proteins were resolved by SDS–PAGE and visualized by autoradiography. Mitochondrial translation profiles for WT, *dpc29*Δ, *mrp7-1* and *dpc29*Δ *mrp7-1* mutant cells are shown in the left autoradiograph, while those for WT, *mrp7-1* and *dpc29*Δ *mrp7-1* mutants deleted for *COB* or *VAR1* are shown in the middle and right autoradiographs, respectively. A segment of corresponding Coomassie stain is shown below each lane. The asterisks indicate the upper and lower novel products observed in *dpc29*Δ *mrp7-1* mutants.

To identify respiratory synthetic lethal mutants, mutagenized *dpc29*Δ cells were screened for the inability to respire following the loss of a plasmid containing wild-type (WT) *DPC29* using an *ADE3/ade2/ade3*-based colony-sectoring assay (24,46,47). Four recessive mutants that fulfilled the criteria of being respiratory synthetically lethal with *dpc29Δ* were placed into a single complementation group, and the corresponding WT gene was cloned from a genomic library. All four mutant alleles were point mutations in the mitoribosomal large subunit protein Mrp7 (bL27) and were designated *mrp7-1*, *mrp7-2*, *mrp7-3* and *mrp7-4* (Figure 4A, B). WT *DPC29* cells containing these mutant alleles formed colonies of equivalent size to the WT on fermentative and respiratory plates at all temperatures tested (Figure 4A; our unpublished results).

The Mrp7 mutations mapped within the same conserved region of the protein. Mrp7 contains both a C-terminal ‘mitospecific’ domain and an N-terminal L27 domain (Figure 4B). This latter domain exhibits high conservation with the prokaryote ribosomal L27 protein (48). All our mutations resided within an unstructured N-terminal stretch of the L27 region that reaches into the peptidyl transferase centre (PTC) of the mitoribosome (Figure 4B) (8). Interestingly, truncations of this extension in *E. coli* impaired growth and decreased peptidyl transferase activity (49–51).

Although the *mrp7 dpc29*Δ respiratory synthetic lethal interaction suggests that our Mrp7 mutant proteins have a direct effect on translation, indirect effects due to protein instability, mislocalization or mitoribosome misassembly are also possible. Localization and steady-state levels of the *mrp7-1* and *mrp7-2* products were determined by comparing whole-cell and mitochondrial lysates (Supplementary Figure S2B). As shown by western analysis, the *mrp7-1* protein migrates at a higher molecular weight than either the *MRP7* or *mrp7-2* products. The *mrp7-1*-encoded protein is probably larger because it contains a point mutation that changes the MTS cleavage residue and prevents N-terminal processing (Supplementary Figure S2B). Thus, this mutant protein retains its MTS following mitochondrial import. The PTC within the mitoribosome is dense with rRNA and protein, therefore retention of the MTS extension on the mature protein could impact its incorporation into the mitoribosome. To assess mitoribosome integrity, we analysed the sedimentation patterns of mitoribosomal proteins in sucrose gradients (24). The mutant *mrp7-1* gene product displayed a similar sedimentation profile to the WT, indicating that the mitoribosomes are stable and contain Mrp7 (Supplementary Figure S2C). Thus, the *mrp7-1* respiratory synthetic lethal phenotype is not due to Mrp7 protein instability, mislocalization or mitoribosome misincorporation.

Given that Mrp7 is a mitoribosomal subunit protein, we asked whether cells bearing mutant *mrp7* alleles would have mitochondrial translation defects. Although the *mrp7* single mutants exhibited a normal *in vivo* mitochondrial translation profile, a minor translation product appeared above Cox2 (Figure 4C; Supplementary Figure S2A). This product was present, along with a band below Atp6, in *mrp7 dpc29Δ* double mutants (Figure 4C; Supplementary Figure S2A). The double mutants also had an overall reduction in mitochondrial translation (Figure 4C; Supplementary Figure S2A). The steady-state mRNA levels in these strains were not reduced (Supplementary Figure S2D), demonstrating that the observed effects occurred during translation. Since all the *mrp7* mutants exhibited similar phenotypes, we limited further experiments to the *mrp7-1* allele.

To determine whether the two novel translation products originated from any of the eight mitochondrial-encoded ORFs, we systematically introduced the *dpc29*Δ and *mrp7* alleles into strains deleted for these ORFs individually and repeated the *in vivo* translation assay. We predicted that loss of a novel translation product following deletion of a single mitochondrial ORF (*COX1*, *COX2*, *COX3*, *COB*, *ATP6*, *ATP8*, *ATP9* or *VAR1*) would indicate that it originated from that gene. Deletion of *VAR1* and *COB* resulted in the loss of the minor higher and lower molecular weight products, respectively (Figure 4C). Thus, these *mrp7* mutants express low levels of truncated *VAR1* and *COB* proteins due to either premature translational termination or internal initiation. These phenotypes are consistent with the idea that mutations within the L27 domain alter the efficiency of the PTC of the mitoribosome (49–51).

### *MRP7* synthetic respiratory lethal mutations reduce cellular cytochrome *c* oxidase activity

**Figure 5.**
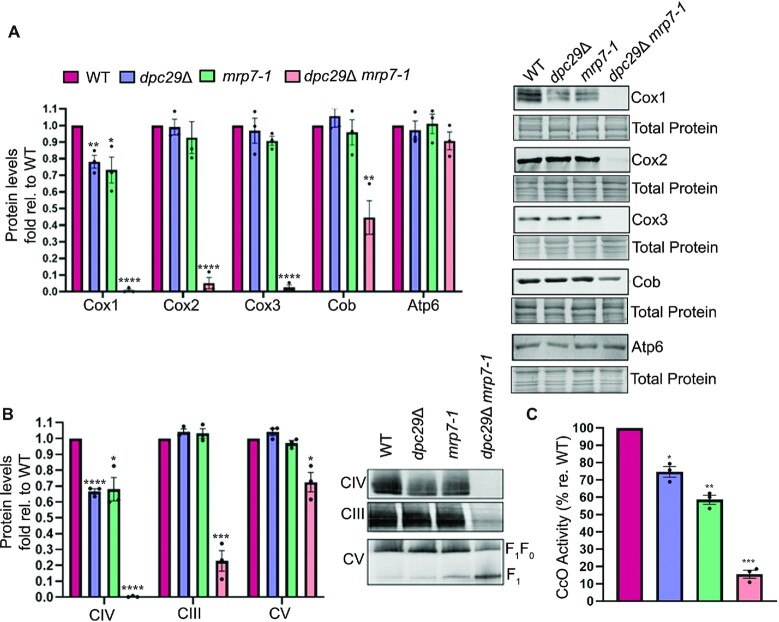
*MRP7* synthetic respiratory lethal mutations reduce cellular cytochrome *c* oxidase activity. (**A**) Steady-state levels of select mitochondrially encoded products. Mitochondrial proteins isolated from WT, *dpc29*Δ, *mrp7-1* or *dpc29*Δ *mrp7-1* cells grown to mid-log phase in galactose were separated by SDS–PAGE, stained, and detected by western blotting (right panel; one of three representative biological repeats). These data were quantified and presented in a colour-coded bar chart (left panel with key above). (**B**) Steady-state levels of OXPHOS complexes. DDM-solubilized mitochondrial respiratory chain complexes were resolved by BN-PAGE and analysed by western blot (right panel; one of three representative biological repeats). The respiratory complexes examined are denoted on the left and the F_1_ subcomplex and fully assembled F_1_F_0_ complex V are labelled on the right. These data were quantified in a bar chart (left panel) using the same colour key as in (A). (**C**) Cytochrome *c* oxidase activity measured in purified mitochondria. Data are shown as a percentage relative activity of WT determined by oxidation of exogenous cytochrome *c* over a 5 min interval. The same key as in (A) is used to denote strains from which mitochondria were isolated. Error bars represent the SE. One, two, three and four asterisks indicate *P*-values <0.05, 0.01, 0.001 and 0.0001, respectively.

Since Cox1 protein levels are modestly reduced in *dpc29*Δ cells, we asked whether our *mrp7* synthetic respiratory lethal mutants shared this phenotype. Steady-state levels of the mitochondrial DNA-encoded proteins were compared by western analysis of isolated mitochondria. Cox1, Cox2 and Cox3 steady-state levels were undetectable in *dpc29*Δ *mrp7-1* double mutants, Cob was 45% of the WT and Atp6 was unaffected (Figure 5A). As expected, decreases in individual complex subunits matched the levels of their corresponding respiratory chain complexes and cytochrome *c* oxidase activity from isolated mitochondria (Figure 5B, C). Analogous to *dpc29*Δ mitochondria, Cox1 levels were reduced in the *mrp7-1* single mutant while the other mitochondrial-encoded proteins tested were unaffected (Figure 5A). Thus, in both *dpc29*Δ and *mrp7* single mutants, Cox1 and complex IV steady-state levels along with cytochrome *c* oxidase activity were reduced while translation appears unaffected as judged by *in vivo* translation assays (Figure 5B, C). As proposed for *dpc29*Δ cells, inefficient *in vivo* translation due to the use of cycloheximide may conceal modest translational defects. Additionally, an excess of mitoribosomes could mask *dpc29*Δ- and *mrp7-1*-induced translational inefficiencies that would otherwise be observed.

### Mitoribosome occupancy of *COX2* and *COB* mRNAs is increased in *mrp7* mutants

**Figure 6.**
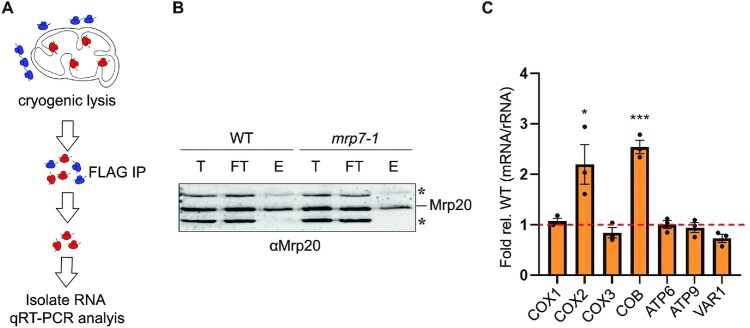
Mitoribosome occupancy is increased in *mrp7* synthetic respiratory lethal mutants. (**A**) Schematic of mitoribosome occupancy assay. Blue and red structures represent cytosolic and mitochondrial ribosomes, respectively. Actively respiring whole cells were subjected to cryogenic lysis, and preserved mitoribosomes containing 3×FLAG-tagged MrpS17 were immunoprecipitated. RNA was isolated from mitoribosomes and mitochondrial RNAs were analysed by qRT-PCR. (**B**) Western analysis of intact mitoribosome immunoprecipitants from WT and *mrp7-1* cells. Total (T), flow-through (FT) and elution (E) fractions from the pulldown were analysed by western blot. Mitoribosome co-immunoprecipitation was verified using antisera directed against the large subunit protein Mrp20. On the right, the Mrp20 protein band is denoted with a line, while non-specific Mrp20 polyclonal antibody interactions are indicated with asterisks. (**C**) Mitoribosome occupancy by mitochondrial mRNAs in *mrp7-1* mutant cells. qRT-PCR analysis was conducted using probes within the coding sequences of *COX1*, *COX2*, *COX3*, *COB*, *ATP6*, *ATP9* and *VAR1*. Bars represent the fold change of each *mrp7-1* mitochondrial mRNA divided by the total *21S* rRNA relative to the WT. Values represent the mean of biological triplicates. Error bars represent the SE. One, two and three asterisks indicate *P*-values <0.05, 0.01 and 0.001, respectively.

To determine whether *mrp7-1* translational defects were masked by an excess of translational capacity, we compared the ratio of translating mitoribosomes in actively growing WT and *mrp7-1* cells. In our approach, cells were cryogenically lysed, and mitoribosomes tagged within the small subunit with MrpS17-3×FLAG were immunoprecipitated (Figure 6A). Intact monosome purification was verified by western blot using antibody reactive against Mrp20, a component of the mitoribosome large subunit (Figure 6B). Both mRNAs and rRNAs were extracted from the purified mitoribosomes and quantified by qRT-PCR. The relative levels of individual mRNAs were then compared with *21S* rRNA from the large subunit. In *mrp7-1* cells, 2.2- and 2.5-fold more mitoribosomes were translating *COX2* and *COB* mRNAs, respectively (Figure 6C). Given that mitoribosomes are in excess and have a higher occupancy level in *mrp7-1* mutants, moderate translational defects may be suppressed. This is consistent with the translational defects reported for the *MRP7* bacterial orthologue *L27* (49–51) and may explain the *dpc29*Δ respiratory synthetic lethal interaction.

### Increased mitoribosome occupancy in *dpc29Δ* cells masks inefficient translation

**Figure 7.**
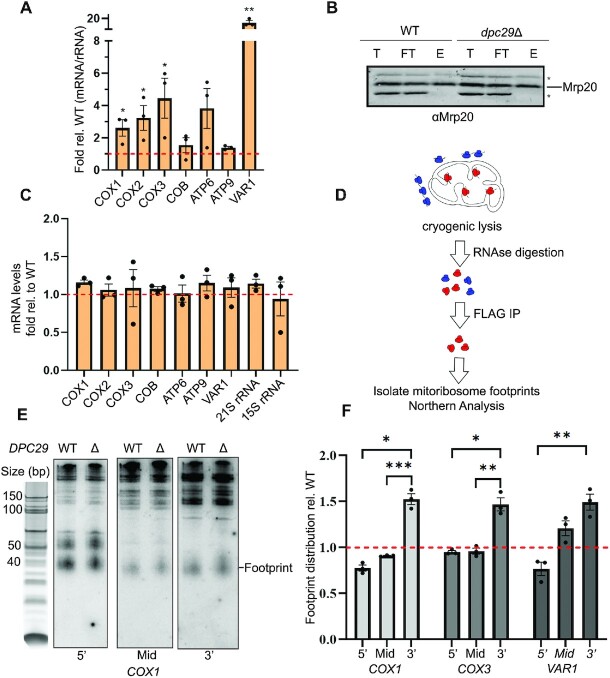
Mitoribosome profiling of *dpc29*Δ cells reveals increased occupancy independent of initiation. (**A**) Mitoribosome occupancy by mitochondrial mRNAs in *dpc29*Δ cells. qRT-PCR analysis was conducted using probes within the coding sequences of *COX1*, *COX2*, *COX3*, *COB*, *ATP6*, *ATP9* and *VAR1*. Bars represent the fold change of each *dpc29*Δ mitochondrial mRNA divided by the total *21S* rRNA relative to WT. (**B**) Western analysis of intact mitoribosome immunoprecipitants from WT and *dpc29*Δ cells. Total (T), flow-through (FT), and elution (E) fractions from the pulldown were analysed by western blot. Mitoribosome co-immunoprecipitation was verified using antisera directed to the large subunit protein Mrp20. On the right, the Mrp20 protein band is denoted with a line, while non-specific Mrp20 polyclonal antibody interactions are indicated with asterisks. (**C**) Steady-state mitochondrial RNA levels in *dpc29*Δ cells. RNA was quantified relative to the WT by qRT-PCR using probes within the coding sequences of *COX1*, *COX2*, *COX3*, *COB*, *ATP6*, *ATP9*, *21S* rRNA and *15S* rRNA. (**D**) Schematic of northern-based mitoribosome profiling assay. Blue and red structures represent cytosolic and mitochondrial ribosomes, respectively. Actively respiring whole cells were subjected to cryogenic lysis and RNase digestion, then preserved mitoribosomes containing 3×FLAG-tagged MrpS17 were immunoprecipitated. RNA footprints were isolated from mitoribosomes and analysed by northern blot. (**E**) Footprint analysis of *COX1*. Protected RNAs isolated from WT (left lane) and *dpc29*Δ (right lane) mitoribosomes were resolved on a denaturing gel and transferred to a nylon membrane. A single blot was stripped and sequentially probed with radiolabelled single-stranded DNA complementing the 5′ (left panel), middle (centre panel) and 3′ (right panel) sequences of the mature *COX1* mRNA. A 10 bp DNA ladder was aligned on the left and the position of the footprint is denoted on the right. (**F**) Footprint distribution within *COX1*, *COX3* and *VAR1* mRNAs in *dpc29*Δ cells. Distribution within each mRNA was determined by quantifying the radioactive signal of the probe bound to the 40 bp footprint using Image Studio. Relative distribution was calculated as a percentage of the total signal of all three probes and then made relative to the WT percentage. All values within this figure were determined from experiments conducted in triplicate. Error bars represent the SE. One, two and three asterisks indicate *P*-values <0.05, 0.01 and 0.001, respectively.

The genetic relationship between *DPC29* and *MRP7* suggested that the *dpc29*Δ cells may also have an excess of mitoribosomes that could compensate for reduced translation efficiency. Mitoribosome occupancy was strongly elevated for five of the seven mRNAs tested, with *COX1*, *COX2*, *COX3*, *ATP6* and *VAR1* increased by 2.6-, 3.2-, 4.5-, 3.8- and 15.4-fold respectively (Figure 7A). These results were independent of transcript levels (Figure 7C).

Evidence suggests that yeast translational activators bind 5′-UTRs of mRNAs and facilitate translation by either resolving inhibitory RNA secondary structures or recruiting mRNAs to the translational apparatus (4,27,52). If Dpc29 was a translational activator, we predicted that mitoribosome profiles of *dpc29*Δ cells would show increased mRNA footprints at the 5′ end. To test this hypothesis, we performed northern-based mitoribosome profiling (35,53) (Figure 7D). This approach was identical to measuring mitoribosome occupancy, except 40 bp mitoribosome footprints were generated and resolved on denaturing urea–acrylamide gels. Northern analysis was then performed using probes to detect the 5′, middle and 3′ ends of the *COX1*, *COX3* and *VAR1* mRNAs. The frequency of the footprints within each of the three regions was quantified as a distribution relative to the total signal across all probes. The footprint distribution was not increased at the 5′ end of any of the mRNAs tested, indicating that the increased mitoribosome mRNA occupancy in *dpc29*Δ cells is not due to reduced translational activation. However, each of the mRNAs tested had a 1.5-fold increased footprint distribution at the 3′ end. This result suggests that the overall translation efficiency of these mRNAs is decreased in *dpc29*Δ cells.

### Human *TACO1* rescues *dpc29* null phenotypes

**Figure 8.**
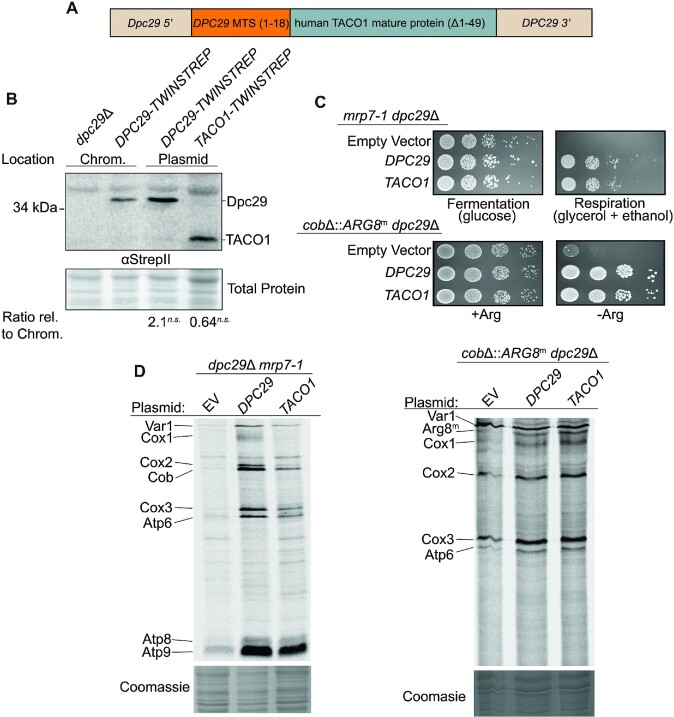
Human *TACO1* rescues *dpc29*Δ phenotypes. (**A**) Construct used for the expression of human *TACO1* in yeast. DNA sequence encoding the 18 amino acid yeast Dpc29 MTS (orange) was fused to a sequence encoding the mature human TACO1 protein (amino acids 50–297) (blue), which was then flanked by the *DPC29* 5′ and 3′ ends (tan). (**B**) Dpc29 and TACO1 expression in yeast. Cells either deleted for *DPC29* or expressing chromosomal *DPC29-TWINSTREP* or a centromeric-plasmid version of *DPC29-TWINSTREP* or *TACO1-TWINSTREP* were grown in galactose media. Whole-cell lysates were prepared, and tagged proteins were analysed by western blot using a StrepII antibody (upper panel) and normalized to the total protein loaded (lower panel). The position of the 34 kDa molecular weight marker is indicated on the left. (**C**) Human *TACO1* rescues *dpc29*Δ *mrp7-1* synthetic respiratory lethal mutants and arginine auxotrophy of the *dpc29*Δ *cox1Δ::ARG8^m^* reporter strain. Top: cells with *dpc29*Δ *mrp7-1* mutations carrying either an empty vector (top row), *DPC29* (middle row) or *TACO1* (bottom row) expression plasmids were grown to mid-log phase in rich glucose media, washed twice, and 1/8 dilutions were spotted onto rich media containing either a fermentative (glucose) or a respiratory (glycerol + ethanol) carbon source and incubated for 3 days at 30°C. Bottom: cells expressing the *cox1Δ*::*ARG8*^m^ reporters and carrying either an empty vector (top row), *DPC29* (middle row) or *TACO1* (bottom row) expression plasmids were grown to mid-log phase in rich glucose media, spotted onto synthetic glucose media containing (+Arg) or lacking (−Arg) arginine and incubated as described above. (**D**) Human *TACO1* complementation of yeast mutant mitochondrial translation profiles. Both *dpc29*Δ *mrp7-1* double mutants (left panel) and the *cobΔ*::*ARG8*^m^ reporter (right panel) grown to mid-log phase in galactose were pulse-labelled with [^35^S]methionine and [^35^S]cysteine in the presence of cycloheximide, and the resulting translation products were resolved by SDS–PAGE and visualized by autoradiography. A segment of corresponding Coomassie stain is shown below each lane. The Arg8^m^ translation product is indicated on the left, directly below the Var1 band. The gel on the right was run 2 h longer to resolve the Arg8^m^ and Cox1 proteins.

Given the strong sequence and structural similarities to Dpc29, we predicted that human TACO1 could function in yeast cells. To express human TACO1 in yeast, the coding sequence of the mature Dpc29 protein was replaced with the corresponding *TACO1* sequence on a single-copy plasmid (Figure 8A). The ability of human TACO1 to replace yeast Dpc29 activity was assayed by two methods. In the first, TACO1 fully rescued the *dpc29*Δ *mrp7-1* respiratory synthetic lethal growth defect and partially rescued the translation defects (Figure 8C, D). In the second, human TACO1 fully rescued the Arg^−^ growth phenotype of the *cox1*Δ::*ARG8*^m^ and *cobΔ::ARG8*^m^ reporters in a *dpc29*Δ null strain (Figure 8C; Supplementary Figure S3B). Human TACO1 partially rescued the *cox1*Δ::*ARG8*^m^ and *cob*Δ::*ARG8*^m^ translation defects in these strains (Figure 8D, right panel; Supplementary Figure S3C). This result indicates that human TACO1 function in yeast is not *COX1* specific. If human TACO1 protein was overexpressed relative to yeast Dpc29, partially functional TACO1 could mimic a full rescue phenotype. To compare TACO1 and Dpc29 levels in yeast cells, both proteins were tagged at the C-terminus with TWINSTREP and their steady-state levels in respiratory media were measured by western blot using the same StrepII antibody (Figure 8B). Protein levels were comparable, demonstrating that the two proteins were equally expressed. These results suggest that *S. cerevisiae* is a useful model organism for studying human TACO1 function.

## DISCUSSION

Due to divergent evolution in eukaryotes, some aspects of mitochondrial gene expression are conserved while others are unique. In *S. cerevisiae*, translational activators are membrane-tethered proteins that orchestrate mitochondrial translation initiation by binding either the elongated 5′-UTRs of their target mRNAs or components of the mitoribosome. However, mammalian mitochondria probably mitochondrially regulate translation by alternative mechanisms since their mRNAs generally lack 5′-UTRs. To date, only one protein has been proposed to act as a gene-specific mitochondrial translational activator in mammals—TACO1. Although this protein was reported to function as a translational activator of *COX1* in humans and mice, its mechanism of action remains unclear. To extend our understanding, we studied the *TACO1* orthologue, *DPC29*, in the yeast model organism. Our experiments show that yeast Dpc29 is a general factor that promotes translation post-initiation, rather than a *COX1* gene-specific translational activator.

Several lines of evidence support the idea that Dpc29 is a general factor that acts at a translational stage post-initiation. First, this role is consistent with experiments where *DPC29* activity alleviated the translational stress of all mtDNA reporters expressing soluble proteins at membrane-directed mitoribosomes. Second, N-terminal mutations within the mitoribosomal protein gene *MRP7* cause a global reduction of mitochondrial translation in *dpc29*Δ cells. Third, northern-based mitoribosome profiling of *dpc29*Δ cells showed higher footprint frequencies at the 3′ end of mRNAs. While human TACO1 only shares 29% identity and 43% similarity with Dpc29, their predicted structures are similar (Figure 1A; Supplementary Figure S1A). Consistent with this observation, TACO1 expression rescues defects in *dpc29*Δ yeast strains, suggesting that the two proteins perform highly conserved functions.

How does human TACO1 functionally complement a *DPC29* deletion in yeast cells if these genes have different proposed functions? Mammalian TACO1 is reported to act as a COX1-specific translational activator, while we provide evidence that yeast Dpc29 acts as a general post-initiation translation factor. One explanation is that they have the same function. TACO1 and *DPC29* deletions in mammals and yeast both produce *COX1*-specific phenotypes upon cursory analysis. Although we show that Dpc29 is a general mitochondrial translation factor, *COX1* expression was most affected by the loss of *DPC29*. We suggest that this could be due to several factors. Cox1 is the most hydrophobic of the mtDNA-encoded proteins, containing 12 transmembrane domains (54). It is also the core enzyme of complex IV and its co-translational insertion into the mitochondrial inner membrane and assembly into cytochrome *c* oxidase is orchestrated by numerous auxiliary factors (12,13,55–63). Thus, it is easy to envisage that inefficient mitochondrial translation elongation would disproportionately affect the synthesis of Cox1. This could make TACO1 function appear COX1 specific. Alternatively, it is possible that some TACO1 and Dpc29 functions overlap, while TACO1 has additional roles in mammalian mitochondria.

Unexpectedly, *VAR1* mitoribosome occupancy in cells lacking *DPC29* was increased at a disproportionately higher rate than the *COX1*, *COX2*, *COX3* or *ATP6* mRNAs. One explanation for this result is that the *VAR1* mRNA is expressed 10- to 50-fold less than the other mitochondrial mRNAs (64), which could cause small changes in occupancy to be amplified. Additionally, other unique Var1 features may explain this result. These include long transcript length, AU-rich mRNA composition, high protein solubility and assembly into the mitoribosome. Longer transcripts may have more potential stall sites, AU-rich mRNAs have more restrictive codon usage, highly soluble proteins may not efficiently transit a mitoribosome exit tunnel optimized for hydrophobic polypeptides, while Var1 synthesis is co-regulated with its assembly into the mitoribosome, which may impact its translational rate (65).

Why is *DPC29* dispensable for respiration in yeast? Why are *dpc29*Δ *mrp7-1* double mutants respiratory synthetic lethal? Although not similar in sequence, do these genes have overlapping functions? Given that human and mouse *TACO1* null mutations cause late-onset Leigh syndrome, we expected that *dpc29*Δ yeast cells would have a respiratory growth phenotype. However, this was not observed, presumably because mitoribosomal subunit proteins in yeast are synthesized in excess to rapidly respond to dynamic cellular demands (66). Additionally, *mrp7* mutants do not exhibit mitochondrial translation defects when *DPC29* is present. These *mrp7* mutations reside within an unstructured N-terminal stretch of the L27 region that reaches into the PTC of the mitoribosome that lead to decreased peptidyl transferase activity when truncated in *E. coli* (44–46). We show that an excess mitoribosome capacity compensates for inefficient translation in *dpc29*Δ and *mrp7-1* single mutants. Currently, the stage of translation at which the *dpc29*Δ and *mrp7-1* mutations have an effect is unclear. However, we believe that the combined translation defects in *dpc29*Δ *mrp7-1* double mutants overwhelm the cell's ability to compensate, resulting in reduced mitochondrial translation, premature termination and respiratory synthetic lethality.

Dpc29 directly interacts with a subset of mitoribosomal proteins during active translation. By incorporating the photoactivable amino acid BPA into Dpc29, we were able to capture protein–protein interactions in real time. Additionally, by selecting specific residues to substitute for BPA, we gained insight into which portions of Dpc29 interact with other proteins. Because there is no crystal structure available for Dpc29, predictive models must be used to interpret these data. Although Dpc29 lacks any recognized motifs, outward-facing residues on both faces of the protein interacted with other proteins. Based on the protein interactants identified, Dpc29 appears to act at two or more different mitoribosome locations: the polypeptide tunnel exit (MrpL8 and MrpL22) and a matrix-facing area near the large subunit central protuberance (MrpL33, MrpL1 and Mrs7). While both regions are important regulatory sites of mitochondrial translation, the nature of these interactions is unclear. Since Dpc29 BPA substitutions were incomplete, additional residue changes may provide a clearer Dpc29–mitoribosome interaction map.

In this report, site-directed photocross-linking of Dpc29 in actively growing cells identified interactions with mitoribosomal proteins not reported by two recent large-scale studies, MIOREX and BioID (10,45). We speculate that Dpc29 was not identified as a component of the MIOREX (mitochondrial organization of gene expression complexes) because these interactome networks were determined by fractionation of mitochondrial lysate fractions that were not actively translating (10). Consistent with this idea, we were also unable to identify any Dpc29 interactions from mitochondrial lysates (our unpublished results). The BioID study found that Dpc29 fused to a biotin ligase (BirA*) biotinylated mitoribosomal subunit proteins, indicating their proximity (45). However, this subset of mitochondrial proteins differed from those identified in this report. This was expected as the labelling radius for BioID is 100 Å (approximately the diameter of the mitoribosome large subunit) (8), while site-directed photocross-linking is zero-length.

The TACO1 protein family is conserved in bacteria and eukaryotes; however, its function is poorly understood. While this study implicates Dpc29 in mitochondrial translation post-initiation, its mechanism of action remains unclear. We speculate that Dpc29 binds mitoribosomes to either resume elongation following a stalling event, stabilize stalled mitoribosomes to prevent premature termination or promote mitoribosome recycling. It is known that hydrophobic proteins with transmembrane domains employ translational stalls to allow for proper folding and co-translational insertion into membranes (67–70). This could explain why Dpc29 and TACO1 mostly affect *COX1*. In contrast, translation of the soluble Arg8^m^ and sfGFP^m^ reporters may be stalled due to steric hindrance of the nascent polypeptide chain. This may result in premature translational termination when Dpc29 is absent. Future experiments employing mitochondrial-encoded reporter genes with engineered stalling as well as performing deep sequencing-based mitoribosome profiling in *dpc29*Δ cells would further define the mechanism by which *DPC29* acts.

## DATA AVAILABILITY

All data generated or analysed during this study are included in this published article (and its supplementary data files).

## Supplementary Material

gkac1229_Supplemental_Files

## Notes

Present address: Michael F. Henry, Molecular Biology, Rowan University Stratford, NJ 08084, USA.

## References

[1] Karlberg O. , Canback B., Kurland C.G., Andersson S.G. The dual origin of the yeast mitochondrial proteome. Yeast. 2000; 17:170–187. 11025528 10.1002/1097-0061(20000930)17:3<170::AID-YEA25>3.0.CO;2-V PMC2448374

[2] Duvezin-Caubet S. , Rak M., Lefebvre-Legendre L., Tetaud E., Bonnefoy N., di Rago J.P. A ‘petite obligate’ mutant of *Saccharomyces cerevisiae*: functional mtDNA is lethal in cells lacking the delta subunit of mitochondrial F1-ATPase. J. Biol. Chem. 2006; 281:16305–16313. 16608846 10.1074/jbc.M513805200

[3] Kirkinezos I.G. , Moraes C.T. Reactive oxygen species and mitochondrial diseases. Semin. Cell Dev. Biol. 2001; 12:449–457. 11735379 10.1006/scdb.2001.0282

[4] Fox T.D. Mitochondrial protein synthesis, import, and assembly. Genetics. 2012; 192:1203–1234. 23212899 10.1534/genetics.112.141267 PMC3512135

[5] Ott M. , Amunts A., Brown A. Organization and regulation of mitochondrial protein synthesis. Annu. Rev. Biochem. 2016; 85:77–101. 26789594 10.1146/annurev-biochem-060815-014334

[6] Masters B.S. , Stohl L.L., Clayton D.A. Yeast mitochondrial RNA polymerase is homologous to those encoded by bacteriophages T3 and T7. Cell. 1987; 51:89–99. 3308116 10.1016/0092-8674(87)90013-4

[7] Jang S.H. , Jaehning J.A. The yeast mitochondrial RNA polymerase specificity factor, MTF1, is similar to bacterial sigma factors. J. Biol. Chem. 1991; 266:22671–22677. 1939277

[8] Desai N. , Brown A., Amunts A., Ramakrishnan V. The structure of the yeast mitochondrial ribosome. Science. 2017; 355:528–531. 28154081 10.1126/science.aal2415 PMC5295643

[9] Haffter P. , McMullin T.W., Fox T.D. A genetic link between an mRNA-specific translational activator and the translation system in yeast mitochondria. Genetics. 1990; 125:495–503. 1696234 10.1093/genetics/125.3.495 PMC1204077

[10] Kehrein K. , Schilling R., Moller-Hergt B.V., Wurm C.A., Jakobs S., Lamkemeyer T., Langer T., Ott M. Organization of mitochondrial gene expression in two distinct ribosome-containing assemblies. Cell Rep. 2015; 10:843–853. 25683707 10.1016/j.celrep.2015.01.012

[11] Jones J.L. , Hofmann K.B., Cowan A.T., Temiakov D., Cramer P., Anikin M. Yeast mitochondrial protein Pet111p binds directly to two distinct targets in COX2 mRNA, suggesting a mechanism of translational activation. J. Biol. Chem. 2019; 294:7528–7536. 30910813 10.1074/jbc.RA118.005355 PMC6509481

[12] Perez-Martinez X. , Broadley S.A., Fox T.D. Mss51p promotes mitochondrial Cox1p synthesis and interacts with newly synthesized Cox1p. EMBO J. 2003; 22:5951–5961. 14592991 10.1093/emboj/cdg566 PMC275423

[13] Shingu-Vazquez M. , Camacho-Villasana Y., Sandoval-Romero L., Butler C.A., Fox T.D., Perez-Martinez X. The carboxyl-terminal end of Cox1 is required for feedback assembly regulation of Cox1 synthesis in *Saccharomyces cerevisiae* mitochondria. J. Biol. Chem. 2010; 285:34382–34389. 20807763 10.1074/jbc.M110.161976 PMC2966052

[14] Gruschke S. , Rompler K., Hildenbeutel M., Kehrein K., Kuhl I., Bonnefoy N., Ott M. The Cbp3–Cbp6 complex coordinates cytochrome b synthesis with bc(1) complex assembly in yeast mitochondria. J. Cell Biol. 2012; 199:137–150. 23007649 10.1083/jcb.201206040 PMC3461508

[15] Christian B.E. , Spremulli L.L. Mechanism of protein biosynthesis in mammalian mitochondria. Biochim. Biophys. Acta. 2012; 1819:1035–1054. 22172991 10.1016/j.bbagrm.2011.11.009 PMC3314146

[16] Weraarpachai W. , Antonicka H., Sasarman F., Seeger J., Schrank B., Kolesar J.E., Lochmuller H., Chevrette M., Kaufman B.A., Horvath R. et al. Mutation in TACO1, encoding a translational activator of COX I, results in cytochrome c oxidase deficiency and late-onset Leigh syndrome. Nat. Genet. 2009; 41:833–837. 19503089 10.1038/ng.390

[17] Richman T.R. , Spahr H., Ermer J.A., Davies S.M., Viola H.M., Bates K.A., Papadimitriou J., Hool L.C., Rodger J., Larsson N.G. et al. Loss of the RNA-binding protein TACO1 causes late-onset mitochondrial dysfunction in mice. Nat. Commun. 2016; 7:11884. 27319982 10.1038/ncomms11884 PMC4915168

[18] Oktay Y. , Gungor S., Zeltner L., Wiethoff S., Schols L., Sonmezler E., Yilmaz E., Munro B., Bender B., Kernstock C. et al. Confirmation of TACO1 as a Leigh syndrome disease gene in two additional families. J. Neuromuscul. Dis. 2020; 7:301–308. 32444556 10.3233/JND-200510 PMC7458500

[19] Morgenstern M. , Stiller S.B., Lubbert P., Peikert C.D., Dannenmaier S., Drepper F., Weill U., Hoss P., Feuerstein R., Gebert M. et al. Definition of a high-confidence mitochondrial proteome at quantitative scale. Cell Rep. 2017; 19:2836–2852. 28658629 10.1016/j.celrep.2017.06.014 PMC5494306

[20] Gietz R.D. , Schiestl R.H., Willems A.R., Woods R.A. Studies on the transformation of intact yeast cells by the LiAc/SS-DNA/PEG procedure. Yeast. 1995; 11:355–360. 7785336 10.1002/yea.320110408

[21] Struhl K. , Stinchcomb D.T., Scherer S., Davis R.W. High-frequency transformation of yeast: autonomous replication of hybrid DNA molecules. Proc. Natl Acad. Sci. USA. 1979; 76:1035–1039. 375221 10.1073/pnas.76.3.1035 PMC383183

[22] Sikorski R.S. , Hieter P. A system of shuttle vectors and yeast host strains designed for efficient manipulation of DNA in *Saccharomyces cerevisiae*. Genetics. 1989; 122:19–27. 2659436 10.1093/genetics/122.1.19 PMC1203683

[23] Schmidt T.G. , Batz L., Bonet L., Carl U., Holzapfel G., Kiem K., Matulewicz K., Niermeier D., Schuchardt I., Stanar K. Development of the Twin-Strep-tag(R) and its application for purification of recombinant proteins from cell culture supernatants. Protein Expr. Purif. 2013; 92:54–61. 24012791 10.1016/j.pep.2013.08.021

[24] Hillman G.A. , Henry M.F. The yeast protein Mam33 functions in the assembly of the mitochondrial ribosome. J. Biol. Chem. 2019; 294:9813–9829. 31053642 10.1074/jbc.RA119.008476 PMC6597815

[25] Laemmli U.K. Cleavage of structural proteins during the assembly of the head of bacteriophage T4. Nature. 1970; 227:680–685. 5432063 10.1038/227680a0

[26] Towbin H. , Staehelin T., Gordon J. Electrophoretic transfer of proteins from polyacrylamide gels to nitrocellulose sheets: procedure and some applications. Proc. Natl Acad. Sci. USA. 1979; 76:4350–4354. 388439 10.1073/pnas.76.9.4350 PMC411572

[27] Steele D.F. , Butler C.A., Fox T.D. Expression of a recoded nuclear gene inserted into yeast mitochondrial DNA is limited by mRNA-specific translational activation. Proc. Natl Acad. Sci. USA. 1996; 93:5253–5257. 8643562 10.1073/pnas.93.11.5253 PMC39231

[28] Bestwick M. , Jeong M.Y., Khalimonchuk O., Kim H., Winge D.R. Analysis of Leigh syndrome mutations in the yeast SURF1 homolog reveals a new member of the cytochrome oxidase assembly factor family. Mol. Cell. Biol. 2010; 30:4480–4491. 20624914 10.1128/MCB.00228-10 PMC2937524

[29] Skrtic M. , Sriskanthadevan S., Jhas B., Gebbia M., Wang X., Wang Z., Hurren R., Jitkova Y., Gronda M., Maclean N. et al. Inhibition of mitochondrial translation as a therapeutic strategy for human acute myeloid leukemia. Cancer Cell. 2011; 20:674–688. 22094260 10.1016/j.ccr.2011.10.015 PMC3221282

[30] Carlstrom A. , Rzepka M., Ott M. The analysis of yeast mitochondrial translation. Methods Mol. Biol. 2021; 2192:227–242. 33230777 10.1007/978-1-0716-0834-0_17

[31] Mootha V.K. , Lepage P., Miller K., Bunkenborg J., Reich M., Hjerrild M., Delmonte T., Villeneuve A., Sladek R., Xu F. et al. Identification of a gene causing human cytochrome c oxidase deficiency by integrative genomics. Proc. Natl Acad. Sci. USA. 2003; 100:605–610. 12529507 10.1073/pnas.242716699 PMC141043

[32] Rak M. , Tzagoloff A. F1-dependent translation of mitochondrially encoded Atp6p and Atp8p subunits of yeast ATP synthase. Proc. Natl Acad. Sci. USA. 2009; 106:18509–18514. 19841266 10.1073/pnas.0910351106 PMC2774022

[33] Pelissier P. , Camougrand N., Velours G., Guerin M. NCA3, a nuclear gene involved in the mitochondrial expression of subunits 6 and 8 of the Fo-F1 ATP synthase of *S. cerevisiae*. Curr. Genet. 1995; 27:409–416. 7586026 10.1007/BF00311209

[34] Shiota T. , Nishikawa S., Endo T. Analyses of protein–protein interactions by in vivo photocrosslinking in budding yeast. Methods Mol. Biol. 2013; 1033:207–217. 23996180 10.1007/978-1-62703-487-6_14

[35] Couvillion M.T. , Churchman L.S. Mitochondrial ribosome (mitoribosome) profiling for monitoring mitochondrial translation in vivo. Curr. Protoc. Mol. Biol. 2017; 119:4.28.21–4.28.25. 10.1002/cpmb.41 PMC5557028 28678443

[36] Kelley L.A. , Mezulis S., Yates C.M., Wass M.N., Sternberg M.J. The Phyre2 web portal for protein modeling, prediction and analysis. Nat. Protoc. 2015; 10:845–858. 25950237 10.1038/nprot.2015.053 PMC5298202

[37] Suhm T. , Habernig L., Rzepka M., Kaimal J.M., Andreasson C., Buttner S., Ott M. A novel system to monitor mitochondrial translation in yeast. Microb. Cell. 2018; 5:158–164. 29487862 10.15698/mic2018.03.621 PMC5826703

[38] Green-Willms N.S. , Butler C.A., Dunstan H.M., Fox T.D. Pet111p, an inner membrane-bound translational activator that limits expression of the *Saccharomyces cerevisiae* mitochondrial gene COX2. J. Biol. Chem. 2001; 276:6392–6397. 11106667 10.1074/jbc.M009856200

[39] Fiori A. , Mason T.L., Fox T.D. Evidence that synthesis of the *Saccharomyces cerevisiae* mitochondrially encoded ribosomal protein Var1p may be membrane localized. Eukaryot. Cell. 2003; 2:651–653. 12796311 10.1128/EC.2.3.651-653.2003 PMC161437

[40] Jia L. , Dienhart M., Schramp M., McCauley M., Hell K., Stuart R.A. Yeast Oxa1 interacts with mitochondrial ribosomes: the importance of the C-terminal region of Oxa1. EMBO J. 2003; 22:6438–6447. 14657017 10.1093/emboj/cdg624 PMC291819

[41] Ott M. , Herrmann J.M. Co-translational membrane insertion of mitochondrially encoded proteins. Biochim. Biophys. Acta. 2010; 1803:767–775. 19962410 10.1016/j.bbamcr.2009.11.010

[42] Ott M. , Prestele M., Bauerschmitt H., Funes S., Bonnefoy N., Herrmann J.M. Mba1, a membrane-associated ribosome receptor in mitochondria. EMBO J. 2006; 25:1603–1610. 16601683 10.1038/sj.emboj.7601070 PMC1440829

[43] Szyrach G. , Ott M., Bonnefoy N., Neupert W., Herrmann J.M. Ribosome binding to the Oxa1 complex facilitates co-translational protein insertion in mitochondria. EMBO J. 2003; 22:6448–6457. 14657018 10.1093/emboj/cdg623 PMC291818

[44] Chin J.W. , Cropp T.A., Chu S., Meggers E., Schultz P.G. Progress toward an expanded eukaryotic genetic code. Chem. Biol. 2003; 10:511–519. 12837384 10.1016/s1074-5521(03)00123-6

[45] Singh A.P. , Salvatori R., Aftab W., Aufschnaiter A., Carlstrom A., Forne I., Imhof A., Ott M. Molecular connectivity of mitochondrial gene expression and OXPHOS biogenesis. Mol. Cell. 2020; 79:1051–1065. 32877643 10.1016/j.molcel.2020.07.024

[46] Bender A. , Pringle J.R. Use of a screen for synthetic lethal and multicopy suppressee mutants to identify two new genes involved in morphogenesis in *Saccharomyces cerevisiae*. Mol. Cell. Biol. 1991; 11:1295–1305. 1996092 10.1128/mcb.11.3.1295-1305.1991 PMC369400

[47] Kranz J.E. , Holm C. Cloning by function: an alternative approach for identifying yeast homologs of genes from other organisms. Proc. Natl Acad. Sci. USA. 1990; 87:6629–6633. 2204059 10.1073/pnas.87.17.6629 PMC54590

[48] Anderson J.M. , Box J.M., Stuart R.A. The mitospecific domain of Mrp7 (bL27) supports mitochondrial translation during fermentation and is required for effective adaptation to respiration. Mol. Biol. Cell. 2022; 33:ar7. 34731012 10.1091/mbc.E21-07-0370 PMC8886811

[49] Maguire B.A. , Beniaminov A.D., Ramu H., Mankin A.S., Zimmermann R.A. A protein component at the heart of an RNA machine: the importance of protein l27 for the function of the bacterial ribosome. Mol. Cell. 2005; 20:427–435. 16285924 10.1016/j.molcel.2005.09.009

[50] Trobro S. , Aqvist J. Role of ribosomal protein L27 in peptidyl transfer. Biochemistry. 2008; 47:4898–4906. 18393533 10.1021/bi8001874

[51] Xiao M. , Wang Y. L27–tRNA interaction revealed by mutagenesis and pH titration. Biophys. Chem. 2012; 167:8–15. 22634088 10.1016/j.bpc.2012.04.003

[52] Herrmann J.M. , Woellhaf M.W., Bonnefoy N. Control of protein synthesis in yeast mitochondria: the concept of translational activators. Biochim. Biophys. Acta. 2013; 1833:286–294. 22450032 10.1016/j.bbamcr.2012.03.007

[53] Couvillion M.T. , Soto I.C., Shipkovenska G., Churchman L.S. Synchronized mitochondrial and cytosolic translation programs. Nature. 2016; 533:499–503. 27225121 10.1038/nature18015 PMC4964289

[54] Garcia-Villegas R. , Camacho-Villasana Y., Shingu-Vazquez M.A., Cabrera-Orefice A., Uribe-Carvajal S., Fox T.D., Perez-Martinez X. The Cox1 C-terminal domain is a central regulator of cytochrome c oxidase biogenesis in yeast mitochondria. J. Biol. Chem. 2017; 292:10912–10925. 28490636 10.1074/jbc.M116.773077 PMC5491776

[55] Manthey G.M. , McEwen J.E. The product of the nuclear gene PET309 is required for translation of mature mRNA and stability or production of intron-containing RNAs derived from the mitochondrial COX1 locus of *Saccharomyces cerevisiae*. EMBO J. 1995; 14:4031–4043. 7664742 10.1002/j.1460-2075.1995.tb00074.x PMC394481

[56] Zambrano A. , Fontanesi F., Solans A., de Oliveira R.L., Fox T.D., Tzagoloff A., Barrientos A. Aberrant translation of cytochrome c oxidase subunit 1 mRNA species in the absence of Mss51p in the yeast *Saccharomyces cerevisiae*. Mol. Biol. Cell. 2007; 18:523–535. 17135289 10.1091/mbc.E06-09-0803 PMC1783774

[57] Mayorga J.P. , Camacho-Villasana Y., Shingu-Vazquez M., Garcia-Villegas R., Zamudio-Ochoa A., Garcia-Guerrero A.E., Hernandez G., Perez-Martinez X. A novel function of Pet54 in regulation of Cox1 synthesis in *Saccharomyces cerevisiae* mitochondria. J. Biol. Chem. 2016; 291:9343–9355. 26929411 10.1074/jbc.M116.721985 PMC4861497

[58] Decoster E. , Simon M., Hatat D., Faye G. The MSS51 gene product is required for the translation of the COX1 mRNA in yeast mitochondria. Mol. Gen. Genet. 1990; 224:111–118. 2177521 10.1007/BF00259457

[59] Barrientos A. , Korr D., Tzagoloff A. Shy1p is necessary for full expression of mitochondrial COX1 in the yeast model of Leigh's syndrome. EMBO J. 2002; 21:43–52. 11782424 10.1093/emboj/21.1.43 PMC125806

[60] Mick D.U. , Wagner K., van der Laan M., Frazier A.E., Perschil I., Pawlas M., Meyer H.E., Warscheid B., Rehling P. Shy1 couples Cox1 translational regulation to cytochrome c oxidase assembly. EMBO J. 2007; 26:4347–4358. 17882259 10.1038/sj.emboj.7601862 PMC2034671

[61] Pierrel F. , Khalimonchuk O., Cobine P.A., Bestwick M., Winge D.R. Coa2 is an assembly factor for yeast cytochrome c oxidase biogenesis that facilitates the maturation of Cox1. Mol. Cell. Biol. 2008; 28:4927–4939. 18541668 10.1128/MCB.00057-08 PMC2519701

[62] Fontanesi F. , Soto I.C., Horn D., Barrientos A. Mss51 and Ssc1 facilitate translational regulation of cytochrome c oxidase biogenesis. Mol. Cell. Biol. 2010; 30:245–259. 19858289 10.1128/MCB.00983-09 PMC2798308

[63] Mick D.U. , Vukotic M., Piechura H., Meyer H.E., Warscheid B., Deckers M., Rehling P. Coa3 and Cox14 are essential for negative feedback regulation of COX1 translation in mitochondria. J. Cell Biol. 2010; 191:141–154. 20876281 10.1083/jcb.201007026 PMC2953447

[64] Turk E.M. , Das V., Seibert R.D., Andrulis E.D. The mitochondrial RNA landscape of *Saccharomyces cerevisiae*. PLoS One. 2013; 8:e78105. 24143261 10.1371/journal.pone.0078105 PMC3797045

[65] Seshadri S.R. , Banarjee C., Barros M.H., Fontanesi F. The translational activator Sov1 coordinates mitochondrial gene expression with mitoribosome biogenesis. Nucleic Acids Res. 2020; 48:6759–6774. 32449921 10.1093/nar/gkaa424 PMC7337963

[66] Bogenhagen D.F. , Ostermeyer-Fay A.G., Haley J.D., Garcia-Diaz M. Kinetics and mechanism of mammalian mitochondrial ribosome assembly. Cell Rep. 2018; 22:1935–1944. 29444443 10.1016/j.celrep.2018.01.066 PMC5855118

[67] Collart M.A. , Weiss B. Ribosome pausing, a dangerous necessity for co-translational events. Nucleic Acids Res. 2020; 48:1043–1055. 31598688 10.1093/nar/gkz763 PMC7026645

[68] Stein K.C. , Frydman J. The stop-and-go traffic regulating protein biogenesis: how translation kinetics controls proteostasis. J. Biol. Chem. 2019; 294:2076–2084. 30504455 10.1074/jbc.REV118.002814 PMC6369277

[69] Zhang G. , Hubalewska M., Ignatova Z. Transient ribosomal attenuation coordinates protein synthesis and co-translational folding. Nat. Struct. Mol. Biol. 2009; 16:274–280. 19198590 10.1038/nsmb.1554

[70] Pechmann S. , Frydman J. Evolutionary conservation of codon optimality reveals hidden signatures of cotranslational folding. Nat. Struct. Mol. Biol. 2013; 20:237–243. 23262490 10.1038/nsmb.2466 PMC3565066

