# Supplementary material for: DPC29 promotes post-initiation mitochondrial translation in Saccharomyces cerevisiae
Source: Nucleic Acids Res. 2023 Jan 9;51(3):1260–76. doi: 10.1093/nar/gkac1229 (PMC9943650; doi:10.1093/nar/gkac1229)
Supplement: gkac1229_Supplemental_Files [file gkac1229_supplemental_files.zip › Supplemental consolidated pdf.pdf]

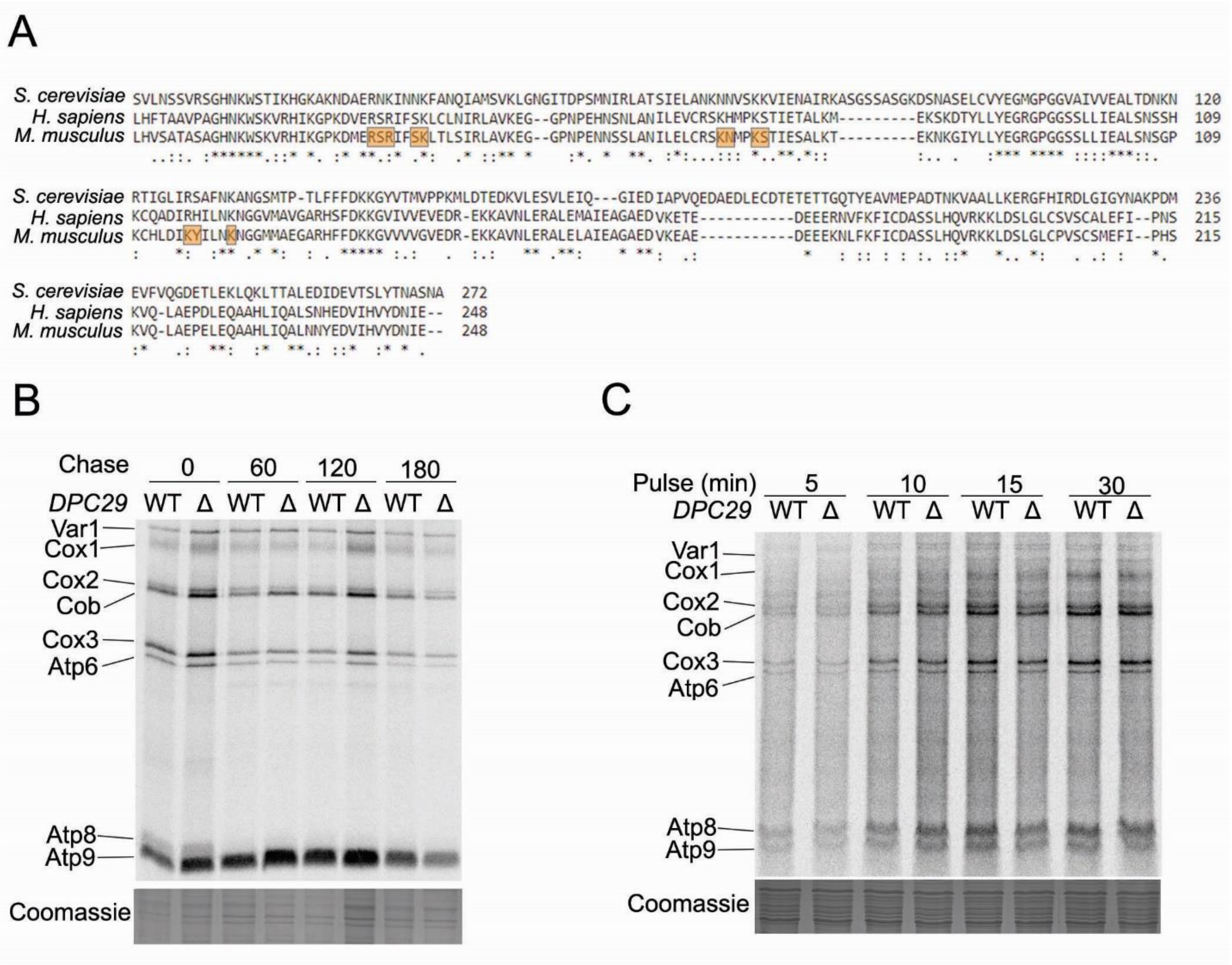

Figure S1. Although *DPC29* is orthologous to human and mouse *TACO1*, Cox1 translation and stability appear *DPC29*-independent by radiolabelling assays (A) An alignment of yeast, human, and mouse mature TACO1 protein sequences generated using Clustal Omega (EMBL-EBI). Asterisks, colons, and periods denote positions that have a fully conserved, strongly similar, and weakly similar residues, respectively. Yellow boxes indicate reported residues required for RNA binding in mouse TACO1. (B) Mitochondrial translation pulse chase profiles. Wildtype (WT) and *dpc29*Δ (Δ) mid-log cells grown in galactose media were pulse-labelled with ^{35}S-methionine and cysteine for 5 min in the presence of cycloheximide and chased with cold methionine before incubation at 37°C. Samples were taken at 0, 60, 120, and 180 minutes. Proteins were resolved by SDS-PAGE and visualized by autoradiography. (C) *In organello* mitochondrial translation pulse profiles. Mitochondria isolated from wildtype (WT) and *dpc29*Δ (Δ) cells grown in galactose were pulse-labelled with ^{35}S-methionine for 5, 10, 15, and 30 min. Solubilized mitochondrial proteins were resolved by SDS-PAGE and visualized by autoradiography. A segment of corresponding Coomassie stain is shown below each lane in (B) and (C).

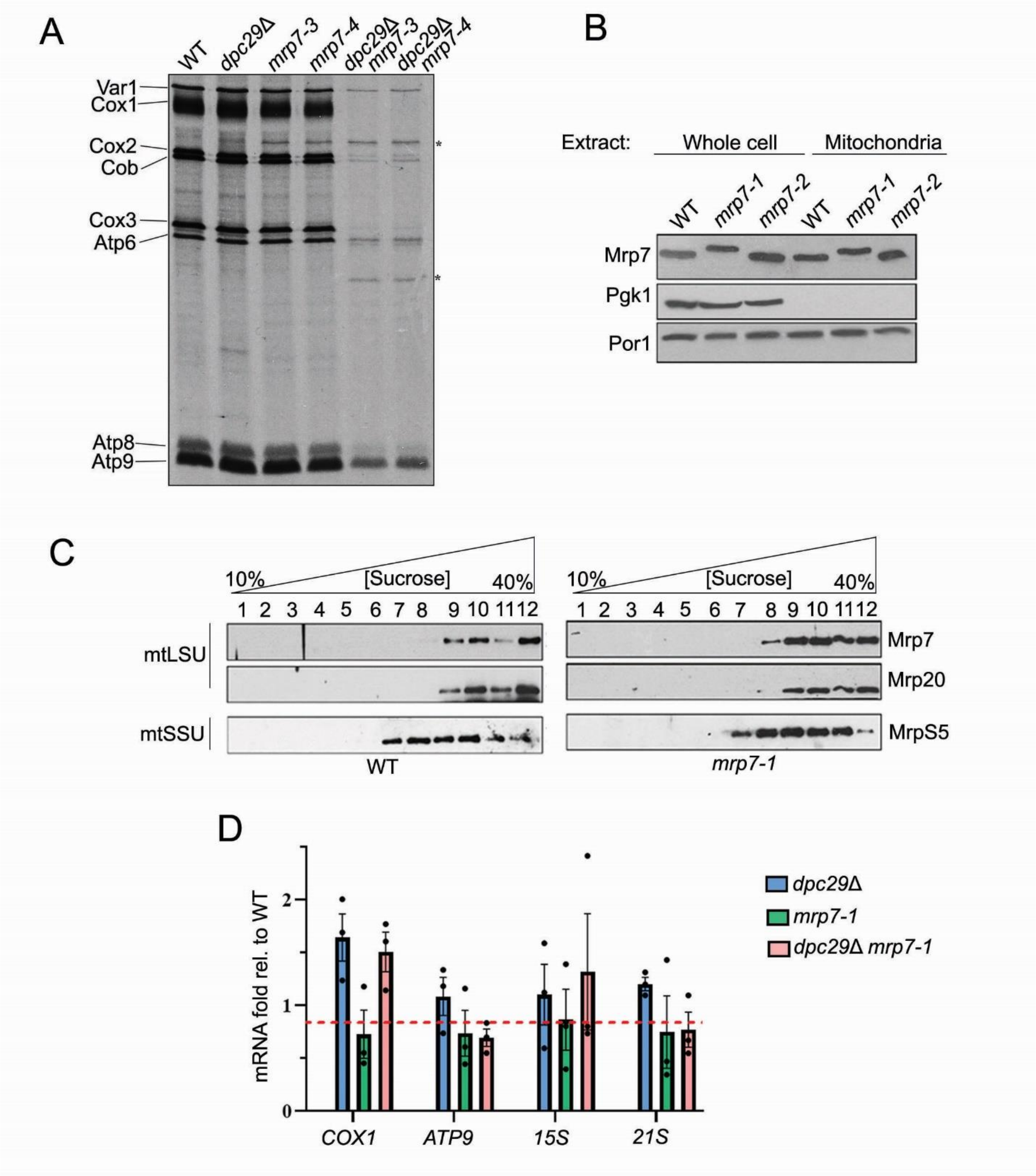

Figure S2. Mrp7 respiratory synthetic lethal mutant proteins are incorporated into mitoribosomes
(A) Mitochondrial translation profiles of WT, *dpc29*∆, *mrp7-3,* and *mrp7-4* single mutants and *dpc29*∆ *mrp7* double respiratory synthetic lethal mutants. Cells were grown to mid-log in galactose and pulse-labelled with ^{35}S-methionine and cysteine in the presence of cycloheximide. Proteins were resolved by SDS-PAGE and visualized by autoradiography. Mitochondrial proteins are denoted on the left. (B) Expression and localization of Mrp7-1 and Mrp7-2 mutant proteins. Whole cell (left three lanes) and isolated mitochondrial extracts (right three lanes) were analyzed by western blot using Mrp7, Pgk1, and

Por1 antibodies (shown on the left). Mitochondrial protein loading was standardized using Por1, while cytosolic Pgk1 confirmed mitochondrial purity. (C) Comparison of mitoribosome integrity in WT and the *mrp7-1* cells. Isolated mitochondria were solubilized and loaded on a 10-40% continuous sucrose gradient, subjected to ultracentrifugation, and separated into twelve fractions. Samples from each fraction were resolved by SDS-PAGE and analyzed by western blot with antibodies for the mitoribosomal large subunit (LSU) proteins Mrp7 and Mrp20, and the small subunit (SSU) protein MrpS5. Mitoribosomal subunits are indicated on the left, while the antibodies used are shown on the right. (D) Steady state RNA levels in *dpc29*Δ (blue), *mrp7-1* (green), and *dpc29*Δ *mrp7-1* (pink) mutants. Quantitative RT-PCR was performed in triplicate on RNA isolated from cells grown in galactose using probes within *COX1*, *ATP9*, *15S* rRNA, and *21S* rRNA.

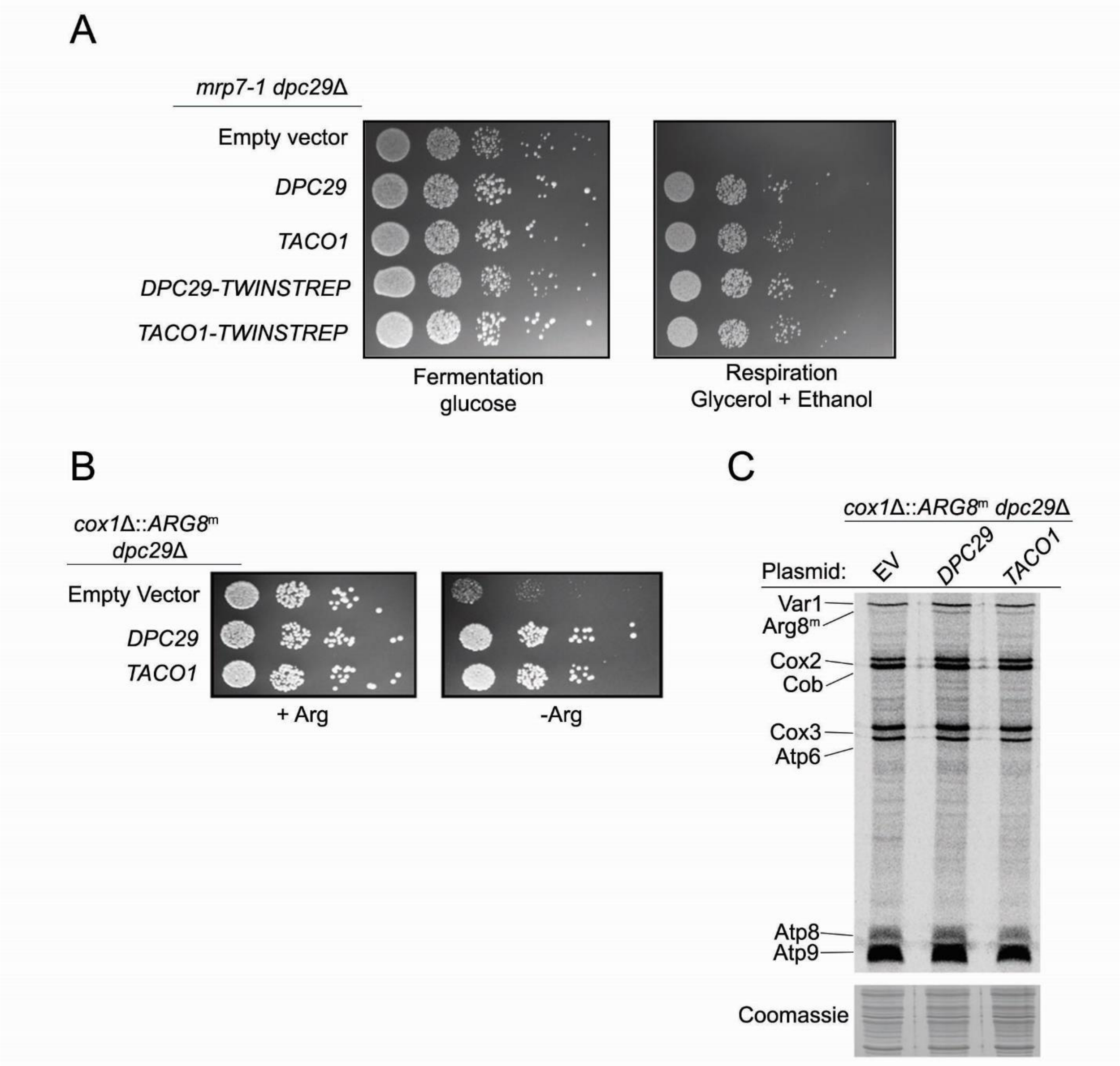

Figure S3. Tagged Human TACO1 and Dpc29 rescues the *dpc29Δ mrp7-1* respiratory growth defect. (A) C-terminal TWINSTREP tagged Dpc29 and TACO1 are functional. Cells with *dpc29Δ mrp7-1* mutations carrying either an empty vector (top row), a *DPC29* expression plasmid (second row), a *TACO1* expression plasmid (third row), *DPC29-TWINSTREP* expression plasmid (fourth row), or *TACO1-TWINSTREP* expression plasmid (fifth row) were grown to mid-log in rich glucose media, washed twice, and 1/8 dilutions were spotted onto rich media containing either a fermentative (glucose) or respiratory (glycerol + ethanol) carbon source and incubated for three days at 30°C. (B) Human TACO1 rescues expression of the *cox1Δ::ARG8*^{m} reporter in a *dpc29Δ* strain. Cells expressing *cox1Δ::ARG8*^{m} reporters and carrying either an empty vector (top row), a *DPC29* expression plasmid (middle row), or a *TACO1* expression plasmid (bottom row) were grown to mid-log in rich glucose media, washed twice, and 1/8 dilutions were spotted onto synthetic glucose media containing (+Arg) or lacking (−Arg) arginine and incubated for three days at 30°C. (C) Complementation of *cox1Δ::ARG8*^{m} reporter mitochondrial translation profile by *TACO1*. Cells expressing *cox1Δ::ARG8*^{m} were grown to mid-log in galactose were pulse-labelled with ^{35}S-methionine and cysteine in the presence of cycloheximide and resulting translation

products were resolved by SDS-PAGE and visualized by autoradiography. A segment of corresponding Coomassie stain is shown below each lane. The Arg8^{m} translation product is indicated on the left, directly below the Var1 band.
